# Intra-host Symbiont Diversity and Extended Symbiont Maintenance in Photosymbiotic Acantharea (Clade F)

**DOI:** 10.3389/fmicb.2018.01998

**Published:** 2018-08-27

**Authors:** Margaret Mars Brisbin, Lisa Y. Mesrop, Mary M. Grossmann, Satoshi Mitarai

**Affiliations:** ^1^Marine Biophysics Unit, Okinawa Institute of Science and Technology Graduate University, Okinawa, Japan; ^2^Department of Biological Sciences, University of Southern California, Los Angeles, CA, United States

**Keywords:** symbiosis, photosymbiosis, plankton, protist, Acantharea, *Phaeocystis*, mutualism, Rhizaria

## Abstract

Photosymbiotic protists contribute to surface primary production in low-nutrient, open-ocean ecosystems and constitute model systems for studying plastid acquisition via endosymbiosis. Little is known, however, about host-symbiont dynamics in these important relationships, and whether these symbioses are mutualistic is debated. In this study, we applied single-cell sequencing methods and advanced fluorescent microscopy to investigate host-symbiont dynamics in clade F acantharians, a major group of photosymbiotic protists in oligotrophic subtropical gyres. We amplified the 18S rRNA gene from single acantharian hosts and environmental samples to assess intra-host symbiont diversity and to determine whether intra-host symbiont community composition directly reflects the available symbiont community in the surrounding environment. Our results demonstrate that clade F acantharians simultaneously host multiple species from the haptophyte genera *Phaeocystis* and *Chrysochromulina*. The intra-host symbiont community composition was distinct from the external free-living symbiont community, suggesting that these acantharians maintain symbionts for extended periods of time. After selectively staining digestive organelles, fluorescent confocal microscopy showed that symbionts were not being systematically digested, which is consistent with extended symbiont maintenance within hosts. Extended maintenance within hosts may benefit symbionts through protection from grazing or viral lysis, and therefore could enhance dispersal, provided that symbionts retain reproductive capacity. The evidence for extended symbiont maintenance therefore allows that *Phaeocystis* could glean some advantage from the symbiosis and leaves the possibility of mutualism.

## Introduction

Photosymbiosis, a nutritional symbiosis where a heterotroph hosts photosynthetic endosymbionts, substantially increases surface primary production in oligotrophic marine ecosystems (Not et al., 2016). Photosymbiosis is also believed to have led to evolution of eukaryotic oxygenic photosynthesis and eventual emergence of diverse photosynthetic eukaryotes, with many evolving from eukaryote-eukaryote secondary and tertiary endosymbiosis (Keeling, 2004). Eukaryote-eukaryote photosymbioses continue to be extremely common among marine protists and contribute significantly to the productivity of oligotrophic open-ocean ecosystems (Decelle et al., 2015). Nonetheless, little is known about host-symbiont dynamics, such as host-symbiont specificity or host mechanisms for symbiont recognition, uptake, and maintenance. Additionally, photosymbioses have traditionally been considered mutualisms under the assumption that hosts provide nitrogen to symbionts and symbionts provide organic carbon to hosts in return (Garcia and Gerardo, 2014). Whether these relationships are truly mutualistic or are instead cases of symbiont exploitation has been increasingly questioned in recent years and there is mounting evidence that exploitation is the rule rather than the exception (Keeling and McCutcheon, 2017). Determining the nature of photosymbioses is particularly interesting, as it could provide insight into how the relationships evolve and persist.

The Acantharea belong to the Rhizaria, a large group of amoeboid protists that includes many photosymbiotic lineages (Burki and Keeling, 2014). Photosymbiotic acantharians are often the most abundant photosymbiotic Rhizaria in oligotrophic surface waters (Michaels et al., 1995), where they contribute significantly to primary production (Caron et al., 1995). The majority of acantharian species (clades E and F) host algal symbionts in the Haptophyte genus *Phaeocystis* (Decelle et al., 2012a). *Phaeocystis* is a globally distributed genus with species that present multiple phenotypes—solitary, flagellated, and colonial—and sometimes form harmful algal blooms (Schoemann et al., 2005). Despite the ecological significance of both partners, this symbiosis remains largely unstudied. There is some evidence, however, suggesting that this relationship is not a case of mutualism and symbionts are instead exploited (Decelle et al., 2012a).

When photosymbiotic protists are cultured in high-light and low-prey conditions, as found in oligotrophic surface waters, hosts benefit from an increased growth-rate, but symbiont growth-rate is suppressed and their photosynthetic efficiency is decreased compared to free-living symbionts (Lowe et al., 2016). These results indicate that algal symbionts may actually experience restricted nitrogen availability *in hospite* and therefore do not benefit from symbiosis (Lowe et al., 2016). Estimated free-living populations of *Phaeocystis* in oligotrophic conditions (Moon-van der Staay et al., 2000) are much larger than possible symbiotic populations estimated from acantharian abundance and symbiont load (Michaels, 1991). The difference in population size between symbiotic and free-living *Phaeocystis* suggests that symbiont growth rate may also be decreased within acantharian hosts, potentially indicating that Acantharea-*Phaeocysti*s photosymbioses are exploitative rather than mutualistic photosymbioses (Decelle, 2013).

Increased growth rate is not the only means by which a symbiont may benefit from a host: decreased predation, viral attack, or competition *in hospite* may allow symbionts to benefit from enhanced dispersal and future reproduction, assuming reproductively viable symbionts are released from hosts (Douglas, 2010; Garcia and Gerardo, 2014). Reproducing symbionts are known to be released from some photosymbiotic protistan hosts: *Chlorella* escapes from *Paramecium* hosts and establishes free-living populations when low-light inhibits host benefit (Lowe et al., 2016) and dinoflagellate symbionts of colonial radiolarians establish free-living populations when isolated from hosts (Probert et al., 2014). Some photosymbiotic forams, however, digest all of their symbionts prior to gametogenesis (Takagi et al., 2016). It is currently unknown whether symbiotic *Phaeocystis* retains reproductive capacity, but symbiotic cells have not yet been cultured from hosts (Decelle et al., 2012a). It is possible that phenotypic changes observed in symbiotic *Phaeocystis*—additional plastids and an enlarged central vacuole (Febvre et al., 1979; Decelle et al., 2012a)—are evidence that symbionts are incapable of cell division, which would make the relationship an ecological and evolutionary dead-end for *Phaeocystis* and preclude the possibility for mutualistm (Decelle et al., 2012a).

The number of symbionts observed in individual acantharians increases with host size (Michaels, 1991), thus requiring that symbionts reproduce *in hospite*, that acantharians recruit new symbionts, or possibly both. If acantharians recruit one or a few symbionts early in development and then support a reproducing symbiont community, the intra-host symbiont community would exhibit low diversity and may be divergent from the free-living environmental community. If symbionts divide within hosts, hosts must exert an alternative form of population control, potentially by shedding (mutualism) (Boettcher et al., 1996; Fishman et al., 2008) or digesting (exploitation) excess symbionts (Titlyanov et al., 1996). Conversely, if acantharians recruit new symbionts, the intra-host symbiont community is likely to be more diverse and representative of the available free-living symbiont community in the surrounding waters. Low-diversity intra-host symbiont communities would therefore suggest that symbionts maintain reproductive capacity and allows for possible symbiont benefit, whereas high-diversity communities may be interpreted as further evidence against mutualism. However, neither intra-host symbiont diversity, nor the relationship between symbiont identity and environmental availability of potential symbionts have been investigated in clade E or F acantharians.

In this study, we applied single-cell Next Generation Sequencing (NGS) to illuminate intra-host symbiont diversity in individual clade F acantharians collected from 7 sampling sites along the Ryukyu Archipelago, spanning more than 1,000 km in the East China Sea (ECS), and near Catalina Island (CA, United States). We further applied NGS to evaluate the environmental availability of symbionts to acantharian hosts sampled from the ECS. We compared molecular symbiont diversity with intra-host symbiont population size by enumerating symbionts with fluorescent confocal microscopy in a subset of acantharians prior to nucleic acid extraction. Additional acantharians were collected and imaged after selectively staining lysosomes and phagolysosomes in order to observe their proximity to symbionts and to determine if symbionts are systematically digested. This study provides new evidence to the mutualism-exploitation debate relative to Acantharea-*Phaeocystis* symbioses by investigating intra-host symbiont diversity and by assessing host-symbiont specificity in the context of environmental symbiont availability.

## Materials and Methods

### Individual Acantharian Sampling

Single acantharians were collected from coastal water near Catalina Island (CA, United States) and from 7 sampling sites along the Ryukyu Archipelago, including coastal water near Okinawa Island (Okinawa, Japan) and from 6 cruise stations visited during the Japan Agency for Marine-Earth Science and Technology (JAMSTEC) MR17-03C cruise to the ECS aboard the R/V *Mirai* in May and June 2017 (**Supplementary Figure S1** and **Supplementary Table S1**). Okinawa Island and Catalina Island plankton samples were collected by pulling a Rigo Simple 20 cm diameter, 100-μm-mesh plankton net or a SEA-GEAR 12^″^ diameter, 163-μm-mesh plankton net, respectively, along the sea surface approximately 5 m behind a small craft at its lowest speed. Aboard the R/V *Mirai*, plankton samples were collected by passing unfiltered seawater pumped from the sea surface through a 100-μm-mesh, hand-held plankton net (Rigo). Plankton samples were observed under a dissecting microscope and individual acantharians were transferred by glass micropipette to clean petri-dishes. Acantharians were rinsed with 0.2-μm-filtered seawater several times until all visible contaminants were removed and then were incubated for 0.5–2 h to allow additional self-cleaning. Acantharians collected aboard the R/V *Mirai* and those from near Okinawa Island were imaged with inverted light microscopy (Zeiss Primovert, Olympus CKX53, **Supplementary Figures S2**, **S3**). Several acantharians collected near Okinawa Island were also imaged with laser confocal microscopy (described below). Each acantharian was transferred to a maximum recovery PCR tube (Axygen) and successful transfer was confirmed by microscopy before adding 30 μL of RLT-plus cell-lysis buffer to each tube (Qiagen). Immediately following buffer addition, samples were flash-frozen with liquid nitrogen and stored at −80°C until later processing in the lab.

### Environmental Sampling

Seawater samples were collected at each ECS cruise station visited by the JAMSTEC MR17-03C cruise where acantharians were also isolated. Two replicates of 4.5 L of seawater were collected from the sea surface by bucket and sequentially filtered under gentle vacuum through 10.0 μm and 0.2 μm pore-size Polytetrafluoroethylene (PTFE) filters (Millipore) to size-fractionate plankton and separate free-living *Phaeocystis* (<10 μm) from acantharian hosts (>10 μm). Filters were flash-frozen in liquid nitrogen onboard and stored at −80°C until processing onshore.

### RNA Extraction From Individual Acantharian Hosts

RNA extractions from single acantharians (*n* = 42, 1–14 per site) were accomplished by modifying methods of Trombetta et al. (2015). Samples were thawed over ice, vortexed twice (10 s, speed 7, Vortex-Genie 2), and then incubated at room temperature for 5 min to fully lyse cells. Agencourt RNAClean XP magnetic beads (Beckman Coulter) were added to each sample at a 2.2:1 V:V ratio and fully mixed by pipette prior to a 30-min incubation in order to bind all RNA to the magnetic beads. After two 80% ethanol washes, RNA was eluted from the beads in 11 μL of a custom elution buffer (10.72 μL nuclease-free water, 0.28 μL RNAase inhibitor (Clonetech)) and 10.5 μL of eluted RNA was further processed following the single-cell protocol for the SMART-seq v4 Ultra Low Input Kit (Clonetech) with 18 cycles in the primary PCR. The resulting cDNA from each sample was quality checked with the Bioanalyzer High Sensitivity DNA Assay (Agilent) and quantified with the Qubit dsDNA High Sensitivity Assay (Qubit 3.0, ThermoFisher).

### DNA Extraction From Environmental Samples

Environmental DNA was extracted from the 0.2-μm pore-size PTFE filters (*n* = 12, 2 replicates from 6 stations) following manufacturer protocols for the Qiagen AllPrep DNA/RNA Mini Kit with limited modifications. Half of each PTFE filter was submerged in RLT-plus cell-lysis buffer (Qiagen) with garnet beads in a 2-μL tube (MoBio/Qiagen). Samples were heated for 10 min at 65°C and then vortexed at maximum speed (Vortex-Genie 2) for 5 min with the MoBio/Qiagen vortex adapter to fully lyse cells. After cell lysis, DNA extraction proceeded without further modifications. Extracted DNA was quantified with the Qubit dsDNA High Sensitivity Assay on a Qubit 3.0 instrument (ThermoFisher).

### Library Preparation and Sequencing

Library preparation for acantharian cDNA samples and environmental DNA samples followed procedures described in the Illumina 16S Metagenomic Sequencing Library Preparation manual, modified only to include universal eukaryotic primers for the V4 region of the eukaryotic 18S rRNA gene (Stoeck et al., 2010) and amplicon PCR conditions most appropriate for these primers. The forward primer, TAReuk454FWD1 (CCAGCASCYGCGGTAATTCC, Stoeck et al., 2010), was used unmodified. The reverse primer, TAReuk454REV3 (ACTTTCGTTCTTGATYRA, Stoeck et al., 2010) was reported to not amplify the *Phaeocystis* 18S gene in *in silico* PCRs (Tanabe et al., 2016). Further investigation revealed a mismatch between the final 3′ adenine in the primer sequence and the *Phaeocystis* 18S gene sequence. Although we found that the original primers do amplify the V4 region of the *Phaeocystis* 18S gene in *de facto* PCRs with DNA extracted from *Phaeocystis* cultures, the mismatch could create bias against *Phaeocystis* sequences in more diverse samples. We therefore eliminated the final 3′ “A” in the TAReuk454REV3 sequence and used a new reverse primer TAReuk454REV3.1 (ACTTTCGTTCTTGATYR). The optimum annealing temperature for the Illumina-adapted primers was determined by performing temperature gradient PCRs (53–65°C, 0.5°C steps) and the annealing step in the amplicon PCR was set at 58°C thereafter. Following the second, indexing PCR and final product purification, amplicon libraries were quantified with the Qubit dsDNA High Sensitivity Assay (Qubit 3.0, ThermoFisher) and the amplicon size was determined with the Bioanalyzer High Sensitivity DNA Assay (Agilent). Amplicon libraries were then submitted to the Okinawa Institute of Science and Technology (OIST) sequencing center for 300 × 300-bp paired-end sequencing on the Illumina MiSeq sequencing platform with v3 chemistry.

### Amplicon Sequence Analysis and Annotation

Demultiplexed paired-end sequences provided by the OIST sequencing center were imported to Qiime2 (v2017.11) software (Caporaso et al., 2010) ^1^. The Divisive Amplicon Denoising Algorithm (DADA) was implemented with the DADA2 plug-in for Qiime2 to perform quality filtering and chimera removal and to construct a feature table consisting of read abundance per amplicon Sequence Variant (SV) by sample (Callahan et al., 2016a). DADA2 models the amplicon sequencing error in order to identify unique SVs and infers sample composition more accurately than traditional Operational Taxonomic Unit (OTU) picking methods that identify representative sequences from clusters of sequences based on a % similarity cut-off (Callahan et al., 2016a). Taxonomy was assigned to SVs in the feature table with a Naïve Bayes classifier trained on SILVA 18S 97% representative sequences and consensus taxonomy (release 128, Quast et al., 2013) using the Qiime2 feature-classifier plug-in (Bokulich et al., 2018). The SV feature table was split into two separate feature tables, one acantharian and one environmental, before both feature tables were extracted from Qiime2 and imported into the R statistical environment (R Core Team, 2013) for further analysis with the R package phyloseq (McMurdie and Holmes, 2013).

Prevalence filtering was applied to both the acantharian and environmental feature tables with phyloseq in order to remove low-prevalence (<5%) SVs and decrease the chance of data artifacts affecting the analysis (Callahan et al., 2016b). Prevalence filtering effectively eliminated most sequences from known Rhizaria prey [i.e., metazoans and diatoms (Swanberg and Caron, 1991)] and parasites (i.e., alveolates, Bråte et al., 2012) from the acantharian feature table. Of the remaining SVs in the acantharian table, 26 were classified as Rhizaria and 21 as Prymnesiophyceae, which includes *Phaeocystis*. In addition, 3 remaining sequences were classified as Holozoa, 2 as Stramenopiles, 2 as Apusomonadidae, 2 as Ancyromonadida, 1 as Chloroplastida, and 5 were not classified. When the unclassified sequences were queried against the GenBank non-redundant nucleotide (nr/nt) database, the top hits for all 5 sequences were Acantharea (BLASTn 2.8.0+, 07/1/2018, Camacho et al., 2009). Unlike Holozoa (metazoans) or Stramenopiles (diatoms), which are acantharian prey (Swanberg and Caron, 1991), Apusomonadidae, Ancyromonidae, and Chloroplastida have not previously been found in association with acantharians. As a result, we considered only the 21 Prymnesiophyceae sequences as symbiotic SVs. In the environmental feature table, there were 187 Prymnesiophyceae sequences remaining after prevalence filtering, but since it is not possible to know which of these can be acantharian symbionts, we further filtered the environmental feature table to only include the 21 symbiotic SVs also found in acantharian samples.

The 21 symbiotic SVs were further classified by building a phylogenetic tree. An initial BLAST query (BLASTn 2.8.0+, 03/23/2018, Camacho et al., 2009) against the GenBank nr/nt database indicated that the symbiont SVs belong to the Haptophyte genera *Phaeocystis* and *Chrysochromulina.* Likewise, a SILVA SSU sequence search (03/23/2018, Quast et al., 2013) classified 18 of the sequences as class Prymnesiophycae, order Phaeocystales or Prymnesiales, and genus *Phaeocystis* or *Chrysochromulina*, when classified to genus level. The remaining 3 symbiotic SVs were designated “unclassified” in the SILVA sequence search. Reference 18S rRNA gene sequences were downloaded from GenBank (Benson et al., 2012) for the 5 Haptophyte orders (Pavlovales, Phaeocystales, Prymnesiales, Isochrysidales, and Coccolithales (Edvardsen and Medlin, 2007) and included in a Multiple Sequence Comparison by Log Expectation (MUSCLE) v3.8.31 (Edgar, 2004) alignment with the 21 symbiotic SVs. A Bayesian phylogenetic tree was then built from the alignment using MrBayes v3.2.7 with the number of nucleotide substitution types (nst) set to 6 (Ronquist and Huelsenbeck, 2003) (**Figure 2**). A phylogenetic tree was built for the 5 dominant acantharian host SVs following the same methods (**Figure 3**) with representative sequences for clades E and F Acantharea (Decelle et al., 2012c) that were also downloaded from GenBank.

### Statistical Analyses

Bray-Curtis distances between symbiont community compositions in acantharian and environmental samples were computed from relative abundances of symbiotic SVs in the filtered feature tables with the R package phyloseq (McMurdie and Holmes, 2013). Bray-Curtis distances were used to perform Principal Coordinate Analyses (PCoA) within the phyloseq package, and PCoA plots were rendered with the R package ggplot2 (Wickham, 2009). Permutational Multivariate Analyses of Variance (PERMANOVA) with 999 permutations were performed with the adonis function in the R package vegan (Oksanen et al., 2013) to determine whether clustering observed in the ordination plots was statistically significant and to discern which covariates were deterministic of symbiont community composition. Specifically, adonis PERMANOVA were performed by location and by host SV on a Bray-Curtis distance matrix including all acantharian samples and by sample type (acantharian or environmental) for a Bray-Curtis distance matrix including the environmental samples and acantharian samples collected at environmental sampling locations. Pairwise PERMANOVA by location and by host SV were also performed on the Bray-Curtis distance matrix including all acantharian samples with the beta-group-significance function in the Qiime2 diversity plugin. Differences were considered statistically significant when the *p*-value was ≤0.05.

### Fluorescent Confocal Microscopy

In order to observe and enumerate symbionts within hosts for which symbiont communities were also evaluated, acantharians collected near Okinawa in April (Oki.3A, Oki.4A) and May (Oki.3, Oki.7, Oki.10, Oki.11) were imaged without staining using an inverted laser scanning confocal microscope (Zeiss LSM 780) prior to RNA extraction. A z-stack of chlorophyll autofluorescence (ex633 nm, em670 nm) and halogen light images was assembled for each host to compare the number of visually observable symbionts to the number of symbiotic SVs identified. In order to evaluate possible host digestion of symbionts, additional acantharian samples were collected in December 2017 (*n* = 3) and stained with LysoTracker Green DND-26 (ThermoFisher), a fluorescent dye that selectively stains acidic compartments (i.e., digestive organelles) within cells, including lysosomes and phagolysosomes. The LysoTracker dye was diluted from the 1-μm stock solution to a 100-nM working solution in 0.2-μm-filtered seawater and each sample was incubated in 100 μL working solution in the dark for 2 hr before imaging. Z-stacks for these samples were assembled by imaging with 3 channels: red for autofluorescence from symbiont chlorophyll (ex633 nm, em670 nm), green for LysoTracker-stained host lysosomes and phagolysosomes (ex488 nm, em514 nm), and gray for halogen light imaging. FIJI Image-J software (Schindelin et al., 2012) was used to adjust image brightness, merge color channels, and create 3-D projections for all imaged host cells.

## Results

### Intra-host Symbiont Diversity in Individual Acantharians

**FIGURE 1:**
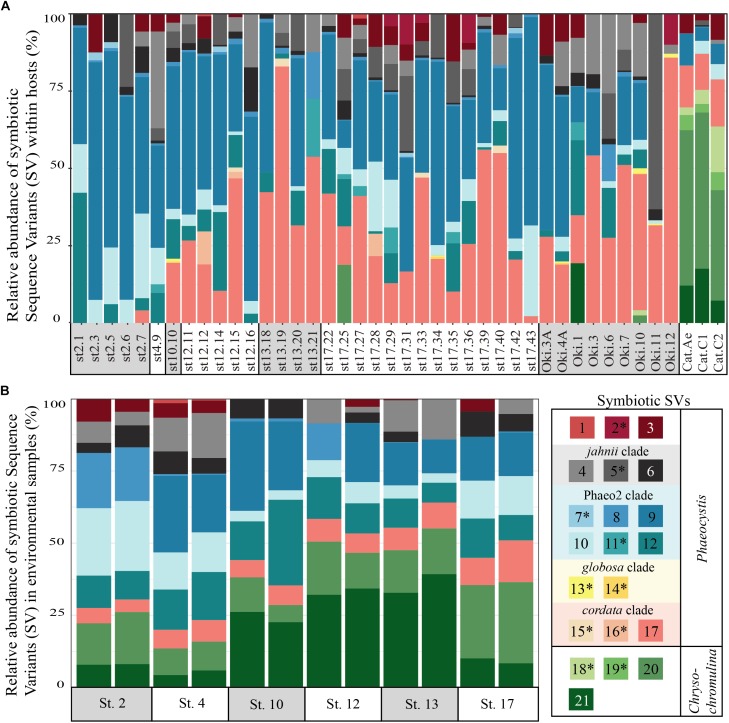
Relative abundance of symbiotic Sequence Variants (SVs) in individual acantharian hosts **(A)** and environmental samples **(B)**. **(A)** Each bar represents a single acantharian host and is labeled by collection location (st#, ECS cruise station number; Oki, Okinawa Island; Cat, Catalina Island) and sample ID. Individual acantharians contained 4–12 symbiotic SVs (mean = 8, *SD* = 2). **(B)** Each bar represents an environmental replicate from ECS cruise stations. Symbiotic SVs are colored by clade: green is *Chrysochromulina*, blue is *Phaeocystis* clade Phaeo2, pink is *Phaeocystis cordata*, gray/black is *Phaeocystis jahnii*, and purple is *Phaeocystis*, but not placed within known clades. Ten of the symbiotic SVs were not found in environmental samples and are marked by an asterisk in the legend. The figure was created with R package ggplot2.

**FIGURE 2:**
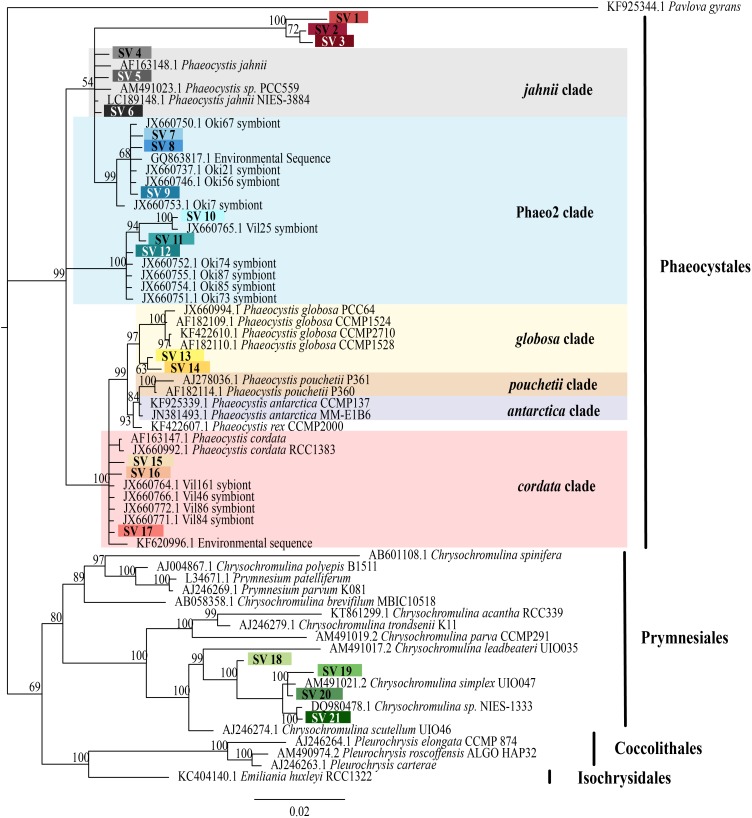
Phylogenetic placement of symbiotic SVs identified from acantharian hosts. The phylogenetic tree was built from a MUSCLE v3.8.31 alignment of 21 symbiotic SVs and GenBank reference sequences representing the 5 haptophyte orders with MrBayes v3.2.7. The symbiotic SVs are highlighted to match the legend in **Figure 1**. *Phaeocystis* clades are color coded and include the Phaeo2 clade, which is an uncultured clade identified from symbiotic sequences from acantharians collected near Okinawa (Decelle et al., 2012b). Values associated with nodes are posterior probabilities as a percent after 100,000 generations (average *SD* of split frequencies = 0.019). The scale bar indicates 0.02 changes expected per site.

To determine if and to what extent intracellular symbiont diversity exists in acantharians, we performed single-cell RNA extractions and sequenced 18S rRNA gene amplicons from 42 individual acantharians. A total of 6,154,808 sequences were generated from acantharian samples, with 28,828–241,809 sequences per sample. After quality filtering and feature table construction, 3,294,093 total sequences remained (5,579–136,339 per sample). Within each acantharian sample, 72–99% of sequences were classified as Rhizaria and 1–17% of sequences were classified as Prymnesiophyceae and therefore designated as deriving from symbionts (**Supplementary Figure S4**). We identified 21 symbiotic SVs in the acantharian samples, and each acantharian host contained 4–12 unique symbiotic SVs (mean = 8, standard deviation = 2) (**Figure 1A**). Our phylogenetic analysis determined that symbiotic SVs belonged to four *Phaeocystis* clades: the *globosa* clade, the *cordata* clade, the *jahnii* clade, which has not previously been reported as a symbiont, and the uncultured Phaeo2 clade, which was discovered by Decelle et al. (2012b) as an acantharian symbiont near Okinawa (**Figure 2**). Additionally, four symbiotic SVs belonged to the genus *Chrysochromulina*, which had not previously been identified as a symbiont in clade F acantharians. The majority of symbiotic SVs in acantharians collected from the ECS belonged to the *Phaeocystis* clades *cordata*, *jahnii* and Phaeo2, and only 3 of these samples contained SVs belonging to *Chrysochromulina*. The opposite pattern was observed in samples collected near Catalina Island: the majority of symbiotic SVs in these samples were *Chrysochromulina* spp. However, all three samples from Catalina Island hosted symbionts from the Phaeo2 *Phaeocystis* clade, which had previously only been found in hosts collected near Okinawa Island (Decelle et al., 2012a). These results demonstrate that acantharians simultaneously host multiple symbiont species and that *Phaeocystis*-hosting acantharians can also host *Chrysochromulina* spp.

**FIGURE 3:**
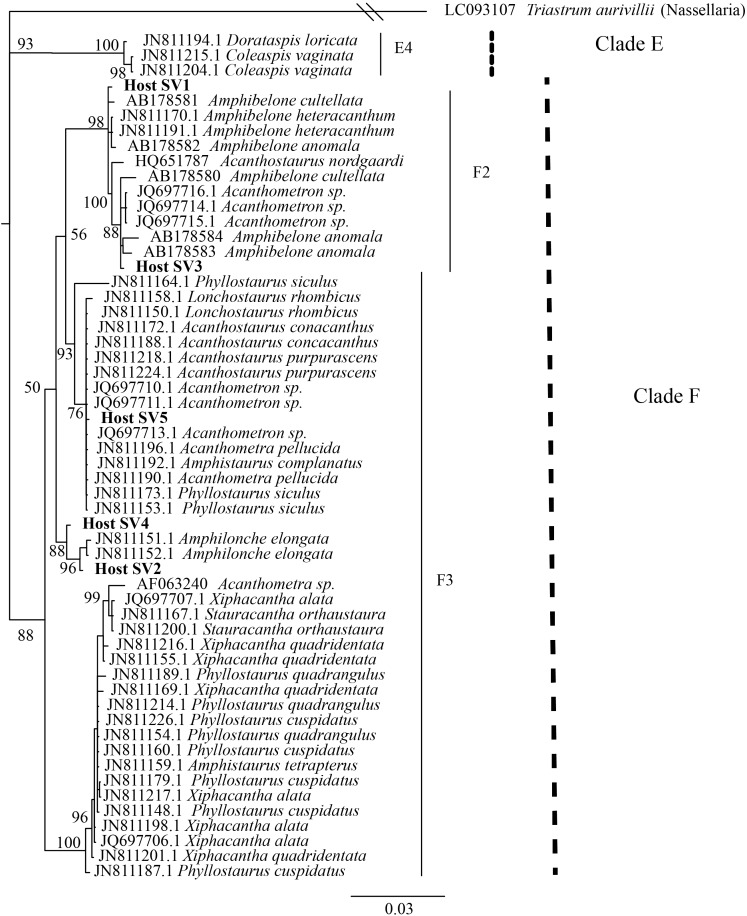
Phylogenetic placement of acantharian host SVs. The phylogenetic tree was built from a MUSCLE v3.8.31 alignment of the 5 dominant host SVs and GenBank reference sequences representing clades E and F Acantharea and the nasellarian radiolarian *Triastrum aurivillii* (outgroup). The tree was built with MrBayes v3.2.7. Values associated with nodes are posterior probabilities as a percent after 40,000 generations (average *SD* of split frequencies = 0.01). The scale bar indicates 0.03 changes expected per site.

**FIGURE 4:**
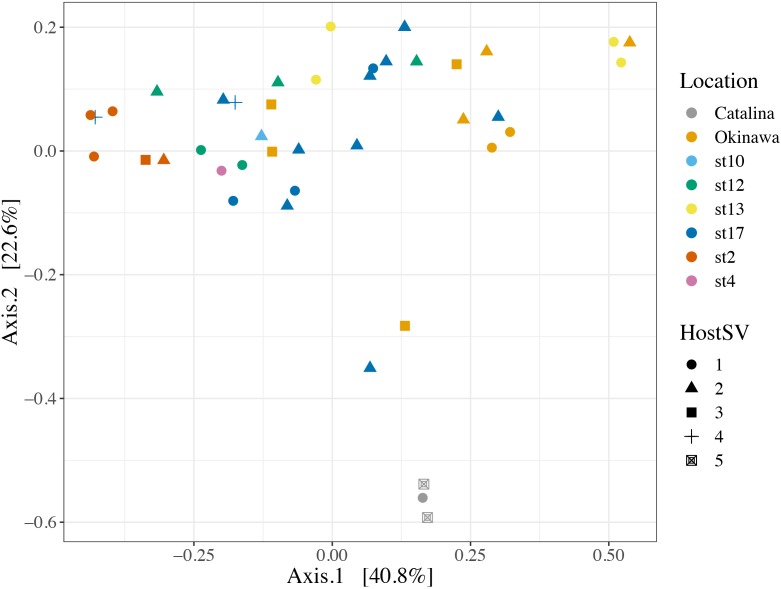
Principal Coordinates Analysis of Bray-Curtis distances between symbiont communities within individual acantharian hosts. Point shape corresponds to host SV and color corresponds to the collection location of the host acantharians (*n* = 42, 1–14 per site). The symbiont communities associated with acantharians collected near Catalina Island and from ECS station 2 form clusters, while communities from the other locations do not cluster separately. A PERMANOVA by location (excluding st. 4 and st. 10 due to insufficient sample size) performed with the adonis function in the R package vegan on the Bray-Curtis distances between symbiont communities confirmed that collection location had a significant effect on symbiont community (*p* = 0.001, *R*^2^ = 0.47 after 999 permutations). Host SV also had a significant effect (*p* = 0.009, *R*^2^ = 0.19), but only SV 5 was significantly different when pairwise PERMANOVA comparisons were performed in Qiime2. Bray-Curtis distance and PCoA ordination were performed with R package phyloseq and the plot was rendered with R package ggplot2.

We also investigated the effect of host type and collection location on symbiont community composition within individual acantharians. Acantharian samples were each dominated by 1 of 5 unique Rhizarian SVs, all of which belonged to Acantharea clade F and represented 3 genera: *Amphibelone* (Host SVs 1 and 3), *Amphilonche* (Host SVs 2 and 4), and *Acanthometra* (Host SV5) (**Figure 3**). Host SV did not appear to determine the symbiont community in a Principal Coordinates Analysis (PCoA) plot based on Bray-Curtis distances between acantharian symbiont community compositions, but collection location did appear to play a role and the Catalina Island samples formed a distinct cluster in the PCoA plot (**Figure 4**). Statistical testing showed that collection location had a significant effect on symbiont community (*p* = 0.001, *R*^2^ = 0.47) and in pairwise comparisons, only two comparisons—st. 17 to st. 12 and st. 13 to Okinawa—were not significantly different (**Supplementary Table S2**). Host SV also significantly affected symbiont community (*p* = 0.009, *R*^2^ = 0.19), but pairwise comparisons showed that the significance was driven solely by the symbiont community associated with host SV 5, which was only found near Catalina Island, indicating that host effect was confounded by location (**Supplementary Table S3**).

### Comparison of Intra-host and Free-Living Symbiont Communities

**FIGURE 5:**
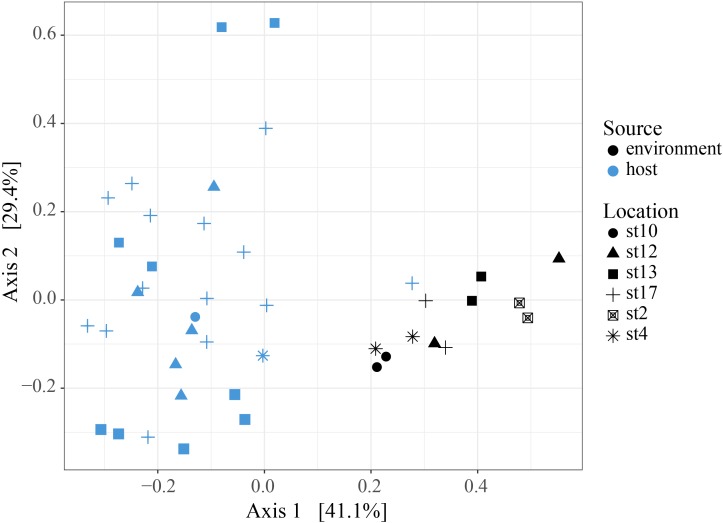
Principal Coordinates Analysis of Bray-Curtis distances between host-associated symbiont communities and free-living symbiont communities. PCoA plot from Bray-Curtis distances between ECS environmental free-living and the host-associated symbiotic community compositions, including all symbiotic SVs identified in this study. The two sample types cluster separately and the difference in community composition is statistically significant (PERMANOVA, *p* = 0.001, *R*^2^ = 0.326, 999 permutations). Analyses were performed with R packages phyloseq and vegan and plot was rendered with R package ggplot2.

To determine whether relative abundance of symbionts within hosts is a reflection of relative abundance of available symbionts in the surrounding water, we compared symbiont communities within acantharians collected from cruise stations in the ECS to environmental samples taken at the same time and place. 1,852,276 sequences were generated from environmental samples with 93,291–246,564 sequences per sample. After quality filtering and feature table construction, 645,325 sequences remained, with 35,691–89,163 sequences per sample. A PCoA plot based on Bray-Curtis distances between the entire community for each environmental sample confirmed that replicates from each location were more similar to each other than to replicates from other locations (**Supplementary Figure S5**). The environmental feature table was subset to include only symbiotic SVs identified in acantharian samples, which comprised 1–3% of sequences in environmental samples. Only 11 of 21 symbiotic SVs identified from acantharian samples were also found in environmental samples (SVs 2, 5, 7, 11, 13, 14, 15, 16, 18, 19 were missing) (**Figure 1B**). *Chrysochromulina* SVs 20 and 21, which were rare in the acantharian samples, were among the most abundant symbiotic SVs in the environment, while *Phaeocystis cordata* SV 17, which was one of the most abundant SVs in acantharian samples, is relatively rare in the environmental samples (**Figures 1A,B**). Intra-host and environmental symbiont communities clustered separately in a PCoA based on Bray-Curtis distances between samples (**Figure 5**) and the observed difference between community compositions in the two sample types was statistically significant (*p* = 0.001, *R*^2^ = 0.33). These results indicate that the intra-host community composition of symbiotic SVs is distinct from the relative availability of symbionts in the surrounding environment.

### Visualization of Host-Associated Symbionts and Host Digestive-Organelles

**FIGURE 6:**
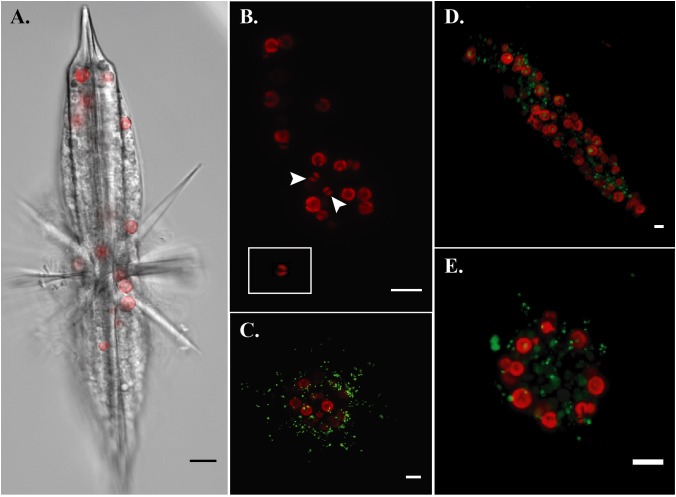
Fluorescent confocal microscopy of acantharians and their symbionts. **(A)** Single optical slice displaying autofluorescence of symbiont chlorophyll (red) and halogen light imaging of an entire host, sample Oki.3A. **(B)** Maximum projection of a z-stack spanning the entire host sample Oki.4A, which contains 17 visible symbionts and 10 symbiotic sequence variants. Arrows indicate symbionts presenting the free-living phenotype: smaller cell diameter and two elongate, parietal chloroplasts, which is also visible in the inset image of a *Phaeocystis globosa* CCMP1528 cell in culture that was imaged following the same methods. Red fluorescence is chlorophyll autofluorescence. **(C–E)** Maximum projections of z-stacks spanning entire hosts collected near Okinawa in December 2017. Red fluorescence is symbiotic chlorophyll autofluorescence. Green fluorescent staining is LysoTracker Green, which selectively binds to low-pH digestive-organelles, including lysosomes and phagolysosomes. Symbionts are not held in phagolysosomes and lysosomes are not concentrated around symbionts, indicating that symbionts are not actively being digested. Scale bars are 10 μm in all panels.

By imaging symbiont chlorophyll autofluorescence with laser confocal microscopy, we were able to clearly enumerate photosynthetic symbionts within hosts. This technique allowed us to discern that there were more individual symbiont cells than observed SVs in each imaged acantharian collected near Okinawa in April and May (**Table 1**, **Figures 6A,B**, and **Supplementary Figure S6**). Symbionts with the free-living phenotype (twin parietal plastids, cell diameter < 5 μm) and symbionts with the symbiotic phenotype (more than 2 plastids, enlarged central vacuole, cell diameter > 5 μm) were observed inside the same host cells (**Figure 6B** and **Supplementary Figure S6**). By selectively staining host digestive-organelles with a fluorescent dye, we were able to visualize host lysosomes and phagolysosomes and their proximity to symbionts (**Figures 6C–E**). We observed lysosomes in both host exoplasm and endoplasm, while symbionts were only observed in host endoplasm. No symbionts were enclosed in phagolysosomes and lysosomes did not appear to be concentrated near symbiont cells.

**Table 1:** Number of acantharian symbionts visible by microscopy and symbiotic SVs observed per host.

	Symbionts visible in micrograph	Symbiotic SVs per host
*Oki.3A*	20	6
*Oki.4A*	17	10
*Oki.3*	29	10
*Oki.7*	38	8
*Oki.10*	28	12
*Oki.11*	24	5

Symbionts within 6 acantharians collected in April (Oki.3A and Oki.4A) and May (Oki.3, Oki.7, Oki.10, Oki.11) from coastal waters near Okinawa, Japan were enumerated by visualizing chlorophyll autofluorescence with laser confocal microscopy. More symbionts were observed in each of the imaged hosts than were detected from analyzing amplicon sequence data.

## Discussion

Acantharians are globally abundant and are especially abundant in low-nutrient subtropical gyres where they contribute to primary production by harboring intra-cellular algal symbionts (Michaels, 1991; Michaels et al., 1995). Despite their ecological importance, host-symbiont dynamics in acantharian photosymbioses remain largely unstudied. Decelle et al. (2012a) discovered that Acantharea-*Phaeocystis* symbioses are flexible in regard to symbiont species and hypothesized that the relationship is more akin to enslavement than mutualism (Decelle et al., 2012a; Decelle, 2013). If symbionts are exploited to the extent that they are unable to reproduce, hosts will need to continuously recruit symbionts and should host diverse symbiont communities reflecting the relative availability of different symbionts. Our amplicon sequencing results show that acantharians do simultaneously host multiple species of *Phaeocystis*, as well as *Chrysochromulina* spp., but we found that the host-associated symbiont community does not simply mirror the free-living community. Our microscopy results show that individual hosts harbor symbionts exhibiting the free-living phenotype as well as the symbiotic phenotype and demonstrate that symbionts are not being systematically digested. Together, these results suggest that hosts recruit symbionts more than once, but that they maintain recruited symbionts.

As acantharians increase in size, the number of symbionts they host also increases (Michaels, 1991). To accomplish this, acantharians could recruit microalgal partners early in development and nurture reproducing symbiont populations. Alternatively, acantharians could recruit more symbionts as they grow, which would likely lead to multiple species of symbionts coexisting within individual hosts. All 42 acantharians collected in this study hosted multiple species of *Phaeocystis* symbionts and several also hosted *Chrysochromulina* spp., indicating that acantharians recruit symbionts more than once. Acantharians in this study also contained more individual symbiont cells than unique Sequence Variants, which could mean that hosts recruit multiple symbiont cells from clonal populations during uptake events, that recruited symbionts divide within hosts, or both. The observation of symbionts with the free-living phenotype alongside symbionts with the symbiotic phenotype within single hosts provides evidence that hosts continue to recruit new symbionts (Febvre et al., 1979, **Figure 6**). Although it is possible that symbionts exhibiting the free-living phenotype are recently divided cells rather than recently engulfed cells, structural changes associated with mitotic division have not been observed in acantharian symbionts (Febvre et al., 1979). These results, however, do not exclude the possibility that symbionts reproduce *in hospite*, and symbionts are known to reproduce within larger photosymbiotic Rhizarians (Takagi et al., 2016).

Since acantharians in this study simultaneously hosted multiple *Phaeocystis* and *Chrysochromulina* species, we expected the intra-host symbiont community to reflect environmental symbiont availability. Our results, however, showed that the relative abundance of symbiotic SVs within hosts was distinct from the relative abundance of these SVs in the surrounding environment. Additionally, acantharians sometimes hosted *Phaeocystis* genotypes (e.g., *P. jahnii* SV 5, *P. globosa* SVs 13 & 14) that were not detected in environmental samples collected from the same place. These results suggest acantharians may maintain symbionts for extended periods, beyond that required for external populations to respond to changing environmental conditions. This is feasible since *Phaeocystis* generation times can be as brief as 6.6 h and vary by species in different light and temperature conditions (Jahnke, 1989), while acantharians likely survive for at least a month (Suzuki and Not, 2015). Intra-host communities may therefore be cumulative representations of all encountered environmental symbiont communities, rather than just a snapshot of the current community.

The observed differences between acantharian symbiont communities and the availability of symbionts could also indicate that hosts selectively uptake symbionts, especially since there were many more prymnesiophyte SVs found in the environment than were identified as symbionts. *Chrysochromulina* SVs were dominant in each acantharian sample collected near Catalina Island, but were only found in one acantharian from an ECS cruise station, despite being well-represented in all cruise station environmental samples. Perhaps *Chrysochromulina* spp. make better partners in the Catalina Island environment compared to the Ryukyu Archipelago. *Chrysochromulina* spp. may also have been much more abundant in the waters near Catalina Island and therefore more likely to become symbionts, since we did not analyze the environmental community there. If acantharians select and concentrate environmentally rare symbionts, it increases the likelihood that our environmental sampling missed those symbionts and provides an alternative explanation as to why some symbiotic SVs were not observed in any environmental samples. It is also possible that the differences observed between intra-host and environmental symbiont communities could derive from different nucleic acid extraction methods used. However, lack of symbiont digestion by hosts provides additional support for extended symbiont maintenance *in hospite*, even if recruitment is highly selective.

This study demonstrates for the first time that intra-cellular symbiont diversity exists in clade F acantharian photosymbioses. Acantharians in the clade B genus*, Acanthochiasma*, also host multiple symbiont types, including *Chrysochromulina* spp. and several dinoflagellate genera (Decelle et al., 2012b). Radiolarian and foraminiferan (Rhizaria) hosts also host dinoflagellates and prasinophytes or prymnesiophytes (Gast and Caron, 2001). Theoretically, simultaneously hosting multiple symbiont species or genotypes is ineffective and should negatively impact host fitness since different symbionts would compete for space and resources within hosts (Douglas, 1998). Planktonic hosts can be transported long distances and may therefore experience larger environmental gradients on shorter time-scales than stationary, benthic photosymbiotic hosts. This could make hosting a diverse community of symbionts more effective for planktonic hosts, especially if some symbionts perform better under different conditions. Indeed, different *Phaeocystis* species have different light and temperature optima (Jahnke, 1989) and different strains of a single species have varying abilities to utilize different nitrogen sources (Wang et al., 2011). Drifting buoys in the Global Drifter Program (Lumpkin et al., 2013) passing our sampling sites in spring, the season we sampled, traveled an average of 3.66° latitude in 30 days (*n* = 42), the estimated minimum survival time of acantharians (Suzuki and Not, 2015), suggesting they can travel at least this far in their lifetime. While the associated mean temperature gradient of 1.93°C is smaller than experimental temperature gradients shown to differentially influence *Phaeocystis* spp., changes in day length and irradiance may affect photosynthetic output of *Phaeocystis* strains differently (Jahnke, 1989).

The possible fitness benefit for symbionts associated with acantharians remains enigmatic, but the evidence we present for extended symbiont maintenance allows that *Phaeocystis* could glean some advantage from the symbiosis. Acantharian symbionts were not enclosed in phagolysosomes in host cells imaged in this study, which could be due to symbionts escaping phagosomes (Jamwal et al., 2016) or a failure of host lysosomes to fuse with symbiont-containing phagosomes (Hohman et al., 1982; Sibley et al., 1985). Electron microscopy suggests that there is a host membrane surrounding acantharian symbionts (Febvre et al., 1979), so it is more probable that lysosomes do not fuse with symbiont-containing phagosomes and they instead represent symbiosomes or long-term perialgal vacuoles. Symbiont signaling prevents lysosomes from fusing with symbiosomes housing *Chlorella* symbionts in *Paramecium* (Kodama et al., 2011) and *Hydra* (Hohman et al., 1982) hosts. Complex signaling between hosts and symbionts leading to development of symbiosomes and symbiont differentiation is suggestive of co-evolution (Hinde and Trautman, 2002). If *Phaeocystis* also actively prevents lysosomes from fusing with phagosomes, then *Phaeocystis* has adapted to avoid digestion and perhaps to promote a stable symbiosis. Although our results suggest that acantharians maintain symbionts, we cannot rule out that they digest symbionts when stressed or before releasing reproductive swarmers, which would rule out mutualism. Acantharian swarmers, which are believed to be gametes, do not contain symbionts and although asexual reproduction has been observed in one group of clade B acantharians, it has not been reported for photosymbiotic acantharians in clades E or F (Decelle and Not, 2015). It is not yet known whether symbionts are digested or released during swarmer production (Decelle and Not, 2015), but nassellarian and spumellarian radiolarians release viable symbionts when producing swarmers (Yuasa and Takahashi, 2016). Additionally, it remains an open question whether released symbionts are reproductively competent outside the host (Decelle et al., 2012a).

Our results demonstrate that clade F acantharians simultaneously associate with multiple *Phaeocystis* and *Chrysochromulina* species, providing further evidence that the Acantharea-*Phaeocystis* photosymbiosis is relatively flexible. Our results suggest that symbionts escape host digestion for extended periods, but whether symbionts are capable of reproducing *in hospite* or after release, and whether they benefit from the relationship is still undetermined. Until acantharians can be maintained for prolonged periods under laboratory conditions, it will remain challenging to elucidate many aspects of the host-symbiont dynamics in this system. LysoTracker dyes can be utilized to track symbiosome conditions for as long as hosts survive and species- or genotype-specific fluorescent probes may be used to investigate whether different species or genotypes are differentially transformed into the symbiotic phenotype or are compartmentalized within the host. If symbionts with a single 18S rRNA gene Sequence Variant (or a unique combination of SVs) are co-localized, it would provide evidence for *in hospite* reproduction. Efforts to culture symbionts from *Phaeocystis*-hosting acantharians should continue. If successful, it would demonstrate unequivocally that symbionts maintain reproductive capacity and cultured symbionts would be an invaluable resource for comparative genomics and transcriptomics. Differential gene expression analysis could then be utilized to investigate physiological shifts in symbiotic *Phaeocystis* or *Chrysochromulina* within hosts compared to free-living cells of symbiotic strains and could illuminate mechanisms of host-symbiont interaction. Further investigation into whether symbionts benefit from this relationship will be important to understanding host-symbiont dynamics in this and other protistan photosymbioses.

## Data Availability Statement

Sequences generated and analyzed in this study have been submitted to the European Nucleotide Archive under the study accession number PRJEB24538. Intermediate data files and data analysis pipelines are available at https://github.com/maggimars/AcanthareaPhotosymbiosis and https://maggimars.github.io/AcanthareaPhotosymbiosis/Analysis.html.

## Author Contributions

MMB, MG, and SM planned the research. MMB performed the research. LM optimized single-cell RNA techniques and performed research. MMB analyzed the data and wrote the manuscript. MG and SM edited the drafts.

## Conflict of Interest Statement

The authors declare that the research was conducted in the absence of any commercial or financial relationships that could be construed as a potential conflict of interest.

## Footnotes

1 qiime2.org

## References

[B1] Benson D. A. Karsch-Mizrachi I. Clark K. Lipman D. J. Ostell J. Sayers E. W. (2012). GenBank. *Nucleic Acids Res.* 40 1–6. 10.1093/nar/gkr1202 22144687 PMC3245039

[B2] Boettcher K. J. Ruby E. G. McFall-Ngai M. J. (1996). Bioluminescence in the symbiotic squid *Euprymna scolopes* is controlled by a daily biological rhythm. *J. Comp. Physiol.* 179 65–73. 10.1007/BF00193435

[B3] Bokulich N. A. Kaehler B. D. Rideout J. R. Dillon M. Bolyen E. Knight R. (2018). Optimizing taxonomic classification of marker-gene amplicon sequences with QIIME 2 ’ s q2 feature-classifier plugin. *Microbiome* 6:90. 10.1186/s40168-018-0470-z 29773078 PMC5956843

[B4] Bråte J. Krabberød A. K. Dolven J. K. Ose R. F. Kristensen T. Bjørklund K. R. (2012). Radiolaria associated with large diversity of marine alveolates. *Protist* 163 767–777. 10.1016/j.protis.2012.04.004 22658831

[B5] Burki F. Keeling P. J. (2014). Rhizaria. *Curr. Biol.* 24 103–107. 10.1016/j.cub.2013.12.025 24502779

[B6] Callahan B. J. McMurdie P. J. Rosen M. J. Han A. W. Johnson A. J. A. Holmes S. P. (2016a). DADA2: high-resolution sample inference from Illumina amplicon data. *Nat. Methods* 13:581. 10.1038/nmeth.3869 27214047 PMC4927377

[B7] Callahan B. J. Sankaran K. Fukuyama J. A. McMurdie P. J. Holmes S. P. (2016b). Bioconductor workflow for microbiome data analysis: from raw reads to community analyses. *F1000Res.* 5:1492. 10.12688/f1000research.8986.2 27508062 PMC4955027

[B8] Camacho C. Coulouris G. Avagyan V. Ma N. Papadopoulos J. Bealer K. (2009). BLAST+: architecture and applications. *BMC Bioinformatics* 10:421 10.1186/1471-2105-10 PMC2803857 20003500

[B9] Caporaso J. G. Kuczynski J. Stombaugh J. Bittinger K. Bushman F. D. Costello E. K. (2010). QIIME allows analysis of high- throughput community sequencing data. *Nat. Methods* 7 335–336. 10.1038/nmeth0510-335 20383131 PMC3156573

[B10] Caron D. A. Michaels A. F. Swanberg N. R. Howse F. A. (1995). Primary productivity by symbiont-bearing planktonic sarcodines (Acantharia, Radiolaria, Foraminifera) in surface waters near bermuda. *J. Plankton Res.* 17 103–129. 10.1093/plankt/17.1.103

[B11] Decelle J. (2013). New perspectives on the functioning and evolution of photosymbiosis in plankton. *Commun. Integr. Biol.* 6:e24560. 10.4161/cib.2456 23986805 PMC3742057

[B12] Decelle J. Colin S. Foster R. A. (2015). “Photosymbiosis in Marine Planktonic Protists,” in *Marine Protists*, ed. Ohtsuka S. (Berlin: Springer), 465–500. 10.1007/978-4-431-55130-0_19

[B13] Decelle J. Not F. (2015). *Acantharia.* Princeton, NJ: ELS 10.1002/9780470015902.a0002102.pub2

[B14] Decelle J. Probert I. Bittner L. Desdevises Y. Colin S. de Vargas C. (2012a). An original mode of symbiosis in open ocean plankton. *PNAS* 109 18000–18005. 10.1073/pnas.1212303109 23071304 PMC3497740

[B15] Decelle J. Siano R. Probert I. Poirier C. Not F. (2012b). Multiple microalgal partners in symbiosis with the acantharian Acanthochiasma sp. (Radiolaria). *Symbiosis* 58 233–244. 10.1007/s13199-012-0195-x

[B16] Decelle J. Suzuki N. Mahé F. de Vargas C. Not F. (2012c). Molecular phylogeny and morphological evolution of the Acantharia (Radiolaria). *Protist* 163 435–450. 10.1016/j.protis.2011.10.002 22154393

[B17] Douglas A. E. (1998). Host benefit and the evolution of specialization in symbiosis. *Heredity* 81 599–603. 10.1046/j.1365-2540.1998.00455.x

[B18] Douglas A. E. (2010). *The Symbiotic Habit.* Princeton, NJ: Princeton University Press.

[B19] Edgar R. C. (2004). Muscle: multiple sequence alignment with high accuracy and high throughput. *Nucleic Acids Res.* 32 1792–1797. 10.1093/nar/gkh340 15034147 PMC390337

[B20] Edvardsen B. Medlin L. K. (2007). “Molecular systematics of Haptophyta,” in *Unravelling the Algae: The Past, Present, and Future of Algal Systematics*, eds Brodie J. Lewis J. (London: CRC Press).

[B21] Febvre J. Febvre-Chevalier C. Ramsay A. T. S. (1979). Ultrastructural study of zooxanthellae of three species of Acantharia (Protozoa, Actinopoda) with details of their taxonomic position in the Prymnesiales (Prymnesiophyceae, Hibbard, 1976). *J. Mar. Biol. Assoc. U. K.* 59 215–226. 10.1017/S0025315400046294

[B22] Fishman Y. Zlotkin E. Sher D. (2008). Expulsion symbiotic algae during feeding by the green hydra - a mechanism for regulating symbiont density? *PLoS One* 3:e2603. 10.1371/journal.pone.0002603 18596972 PMC2432043

[B23] Garcia J. R. Gerardo N. M. (2014). The symbiont side of symbiosis: do microbes really benefit? *Front. Microbiol.* 5:510. 10.3389/fmicb.2014.00510 25309530 PMC4176458

[B24] Gast R. J. Caron D. A. (2001). Photosymbiotic associations in planktonic foraminifera and radiolaria. *Hydrobiologia* 461 1–7. 10.1023/A:1012710909023

[B25] Hinde R. Trautman D. (2002). “Symbiosomes,” in *Symbiosis: Mechanisms and Model Systems* Vol. 4 ed. Seckbach J. (Springer Science and Business Media).

[B26] Hohman T. C. McNeil P. L. Muscatine L. (1982). Phagosome-lysosome fusion inhibited by algal symbionts of hydra Viridis. *J. Cell Biol.* 94 56–63. 10.1083/jcb.94.1.56 7119017 PMC2112179

[B27] Jahnke J. (1989). The light and temperature dependence of growth rate and elemental composition of *Phaeocystis globosa* (Scherffel) and *Phaeocystis pouchetii* (Har.) lagerh. in batch cultures. Netherlands. *J. Sea Res.* 23 15–21. 10.1016/0077-7579(89)90038-0

[B28] Jamwal S. V. Mehrotra P. Singh A. Siddiqui Z. Basu A. Rao K. V. S. (2016). Mycobacterial escape from macrophage phagosomes to the cytoplasm represents an alternate adaptation mechanism. *Sci. Rep.* 6 1–9. 10.1038/srep23089 26980157 PMC4793295

[B29] Keeling P. J. (2004). Diversity and evolutionary history of plastids and their hosts. *Am. J. Bot.* 91 1481–1493. 10.3732/ajb.91.10.1481 21652304

[B30] Keeling P. J. McCutcheon J. P. (2017). Endosymbiosis: the feeling is not mutual. *J. Theor. Biol.* 0 1–5. 10.1016/j.jtbi.2017.06.008 28624393 PMC5772595

[B31] Kodama Y. Inouye I. Fujishima M. (2011). Symbiotic *Chlorella vulgaris* of the ciliate *Paramecium bursaria* plays an important role in maintaining perialgal vacuole membrane functions. *Protist* 162 288–303. 10.1016/j.protis.2010.06.005 20884291

[B32] Lowe C. D. Minter E. J. Cameron D. D. Brockhurst M. A. (2016). Shining a light on exploitative host control in a photosynthetic endosymbiosis. *Curr. Biol.* 26 207–211. 10.1016/j.cub.2015.11.052 26748854

[B33] Lumpkin R. Grodsky S. A. Centurioni L. Rio M. H. Carton J. A. Lee D. (2013). Removing spurious low-frequency variability in drifter velocities. *J. Atmos. Ocean. Technol.* 30 353–360. 10.1175/JTECH-D-12-00139.1

[B34] McMurdie P. J. Holmes S. (2013). Phyloseq: an r package for reproducible interactive analysis and graphics of microbiome census data. *PLoS One* 8:e61217. 10.1371/journal.pone.0061217 23630581 PMC3632530

[B35] Michaels A. F. (1991). Acantharian abundance and symbiont productivity at the VERTEX seasonal station. *J. Plankton Res.* 13 399–418. 10.1093/plankt/13.2.399

[B36] Michaels A. F. Caron D. A. Swanberg N. R. Howse F. A. Michaels C. M. (1995). Planktonic sarcodines (Acantharia, Radiolaria, Foraminifera) in surface waters near Bermuda: abundance, biomass and vertical flux. *J. Plankton Res.* 17 131–163. 10.1093/plankt/17.1.131

[B37] Moon-van der Staay S. Y. van der Staay G. W. M. Guillou L. Vaulot D. Claustre H. Medlin L. K. (2000). Abundance and diversity of prymnesiophytes in the picoplankton community from the equatorial Pacific Ocean inferred from 18S rDNA sequences. *Limnol. Oceanogr.* 45 98–109. 10.4319/lo.2000.45.1.0098

[B38] Not F. Probert I. Ribeiro C. Crenn K. Guillou L. Jeanthon C. (2016). “Photosymbiosis in the marine pelagic environments,” in *The Marine Microbiome*, ed. Cretoiu M. S. (New York, NY: Springer International Publishing).

[B39] Oksanen J. Blanchet F. G. Kindt R. Legendre P. Minchin P. R. O’hara R. B. (2013). Package ‘vegan.’. *Commun. Ecol. Packag.* 9:2.

[B40] Probert I. Siano R. Poirier C. Decelle J. Biard T. Tuji A. (2014). *Brandtodinium* gen. nov. and *B.nutricula* comb. Nov. (Dinophyceae), a dinoflagellate commonly found in symbiosis with polycystine radiolarians. *J. Phycol.* 50 388–399. 10.1111/jpy.12174 26988195

[B41] Quast C. Pruesse E. Yilmaz P. Gerken J. Schweer T. Glo F. O. (2013). The silva ribosomal RNA gene database project: improved data processing and web-based tools. *Nucleic Acids Res.* 41 590–596. 10.1093/nar/gks1219 23193283 PMC3531112

[B42] R Core Team. (2013). *R: A Language and Environment for Statistical Computing.* Vienna: R Foundation for Statistical Computing.

[B43] Ronquist F. Huelsenbeck J. P. (2003). MrBayes 3: Bayesian phylogenetic inference under mixed models. *Bioinformatics* 19 1572–1574. 10.1093/bioinformatics/btg180 12912839

[B44] Schindelin J. Arganda-Carreras I. Frise E. Kaynig V. Longair M. Pietzsch T. (2012). Fiji: an open-source platform for biological-image analysis. *Nat. Methods* 9 676–682. 10.1038/nmeth.2019 22743772 PMC3855844

[B45] Schoemann V. Becquevort S. Stefels J. Rousseau V. Lancelot C. (2005). *Phaeocystis* blooms in the global ocean and their controlling mechanisms: a review. *J. Sea Res.* 53 43–66. 10.1016/j.seares.2004.01.008

[B46] Sibley L. D. Weidner E. Krahenbuhl J. L. (1985). Phagosome acidification blocked by intracellular *Toxoplasma gondii*. *Nature* 315 416–419. 10.1038/315416a0 2860567

[B47] Stoeck T. Bass D. Nebel M. Christen R. Jones M. D. M. Breiner H. W. (2010). Multiple marker parallel tag environmental DNA sequencing reveals a highly complex eukaryotic community in marine anoxic water. *Mol. Ecol.* 19 21–31. 10.1111/j.1365-294X.2009.04480.x 20331767

[B48] Suzuki N. Not F. (2015). “Biology and ecology of Radiolaria,” in *Marine Protists*, ed. Not N. (Berlin: Springer), 179–222. 10.1007/978-4-431-55130-0_8

[B49] Swanberg N. R. Caron D. A. (1991). Patterns of sarcodine feeding in epipelagic oceanic plankton. *J. Plankton Res.* 13 287–312. 10.1093/plankt/13.2.287

[B50] Takagi H. Kimoto K. Fujiki T. Kurasawa A. Moriya K. Hirano H. (2016). Ontogenetic dynamics of photosymbiosis in cultured planktic foraminifers revealed by fast repetition rate fluorometry. *Mar. Micropaleontol.* 122 44–52. 10.1016/j.marmicro.2015.10.003

[B51] Tanabe A. S. Nagai S. Hida K. Yasuike M. Fujiwara A. Nakamura Y. (2016). Comparative study of the validity of three regions of the 18S-rRNA gene for massively parallel sequencing-based monitoring of the planktonic eukaryote community. *Mol. Ecol. Resour.* 16 402–414. 10.1111/1755-0998.12459 26309223

[B52] Titlyanov E. A. Titlyanov T. V. Leletkin V. A. Tsukahaea J. van Woesik R. Yamazato K. (1996). Degradation of zooxanthellae and regulation of their density in hermatypic corals. *Mar. Ecol. Prog. Ser.* 139 167–178. 10.3354/meps139167

[B53] Trombetta J. J. Gennert D. Lu D. Satija R. Shalek A. K. Regev A. (2015). Preparation of single-cell RNA-seq libraries for next generation sequencing. *Curr. Protoc. Mol. Biol.* 107 1–25. 10.1002/0471142727.mb0422s107 24984854 PMC4338574

[B54] Wang X. Wang Y. Smith W. O. (2011). The role of nitrogen on the growth and colony development of *Phaeocystis globosa* (Prymnesiophyceae). *Eur. J. Phycol.* 46 305–314. 10.1080/09670262.2011.602430

[B55] Wickham H. (2009). *Ggplot2: Elegant Graphics for Data Analysis.* New York, NY: Springer-Verlag 10.1007/978-0-387-98141-3

[B56] Yuasa T. Takahashi O. (2016). Light and electron microscopic observations of the reproductive swarmer cells of nassellarian and spumellarian polycystines (Radiolaria). *Eur. J. Protistol.* 54 19–32. 10.1016/j.ejop.2016.02.007 27023270

