# Supplementary material for: Intra-host Symbiont Diversity and Extended Symbiont Maintenance in Photosymbiotic Acantharea (Clade F)
Source: Front Microbiol. 2018 Aug 27;9:1998. doi: 10.3389/fmicb.2018.01998 (PMC6120437; doi:10.3389/fmicb.2018.01998)
Supplement: Supplementary file 1 [file Data_Sheet_1.PDF]

Supplementary Material

Intra-host symbiont diversity and extended symbiont maintenance in photosymbiotic Acantharea (clade F)

Margaret Mars Brisbin*, Lisa Y. Mesrop, Mary M. Grossmann, Satoshi Mitarai

*** Correspondence:** margaret.marsbrisbin@oist.jp

1 Supplementary Data

Intermediate data files and data analysis pipelines are available at https://github.com/maggimars/AcanthareaPhotosymbiosis and https://maggimars.github.io/AcanthareaPhotosymbiosis/Analysis.html.

2 Supplementary Figures and Tables

2.1 Supplementary Figures

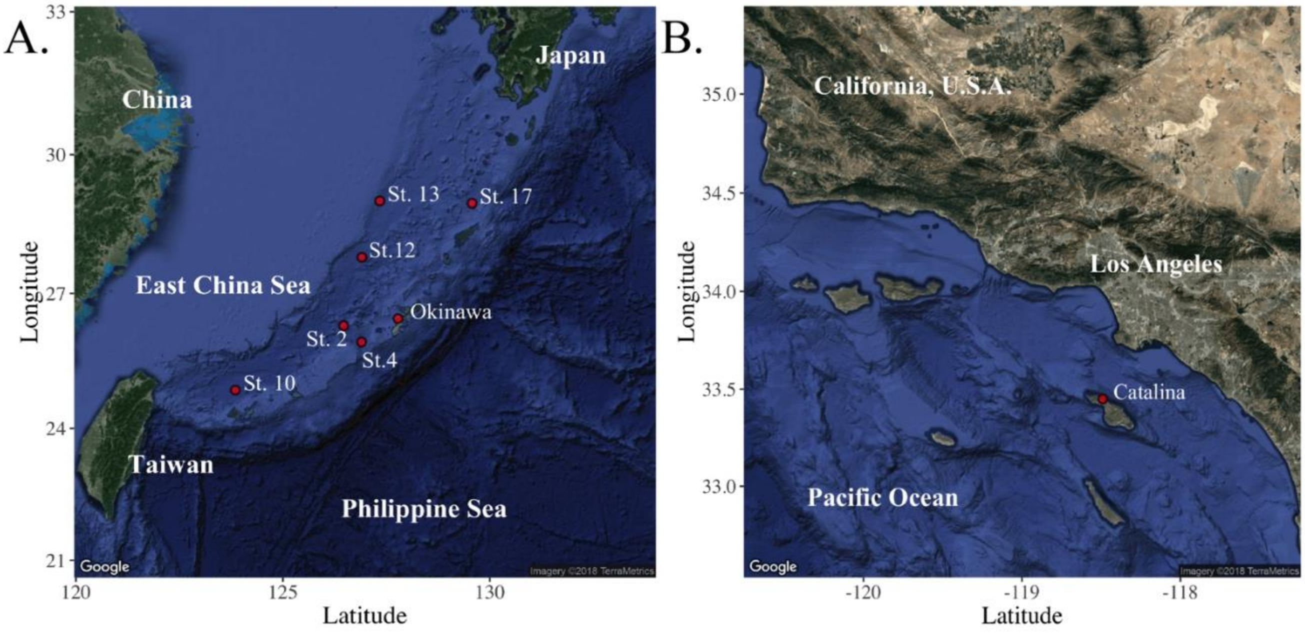

Supplemental Figure S1. Sampling sites along the Ryukyu Archipelago in the East China Sea (ECS) (A) and near Catalina Island, California, U.S.A. (B). (A) ECS Stations 2, 4, 10, 12, 13, and 17 were sampled during the Japan Agency for Marine-Earth Science and Technology (JAMSTEC) MR17-03C cruise in May and June 2017. Samples were collected from the Okinawa Island (Okinawa, Japan) sampling site in April, May, and December 2017. (B) Additional samples were collected near the University of Southern California's Wrigley Institute for Environmental Studies on Catalina Island, California, U.S.A. in May 2017. Maps were rendered with the R package ggmap using Google satellite data.

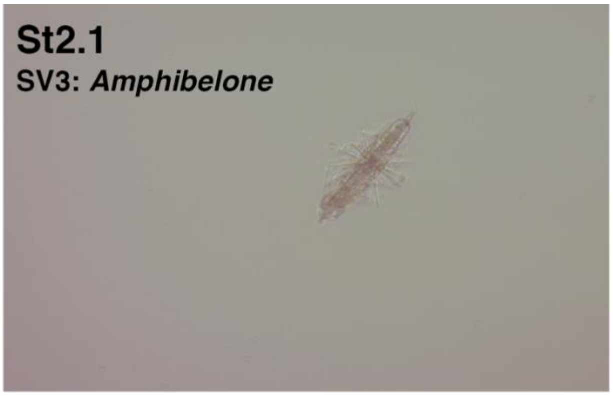
St2.1
SV3: *Amphibelone*

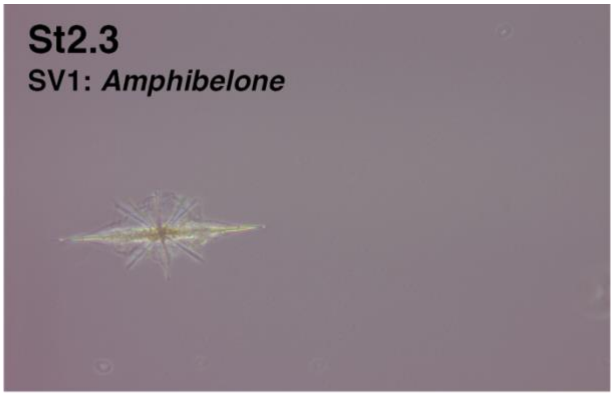
St2.3
SV1: *Amphibelone*

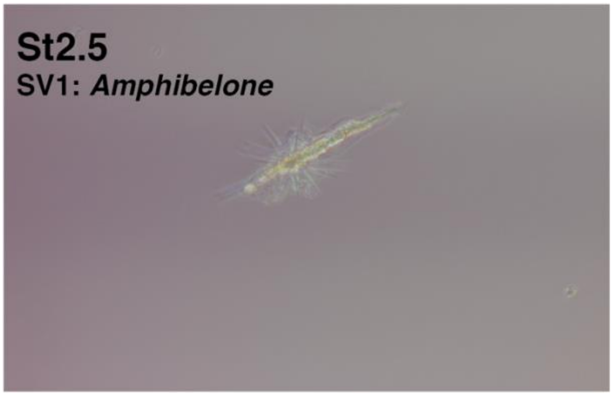
St2.5
SV1: *Amphibelone*

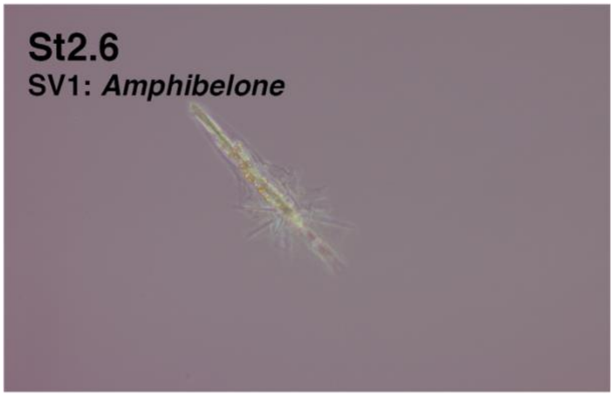
St2.6
SV1: *Amphibelone*

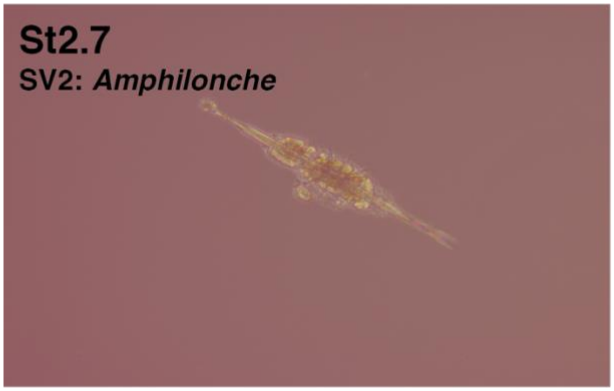
St2.7
SV2: *Amphilonche*

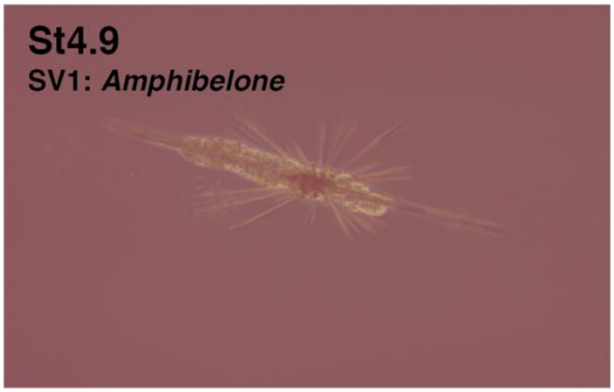
St4.9
SV1: *Amphibelone*

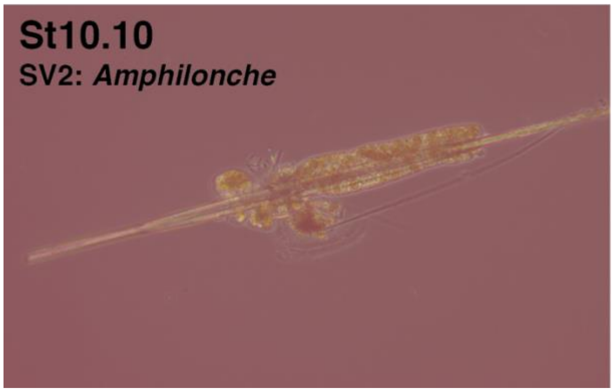
St10.10
SV2: *Amphilonche*

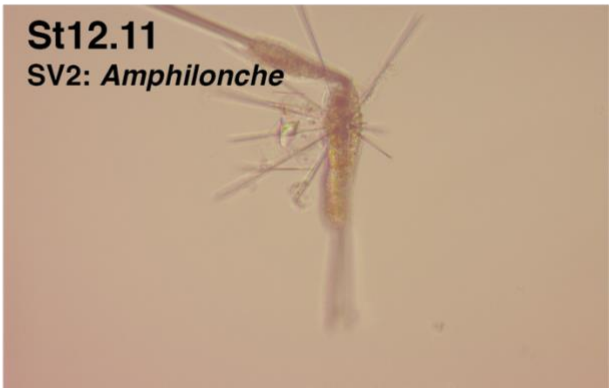
St12.11
SV2: *Amphilonche*

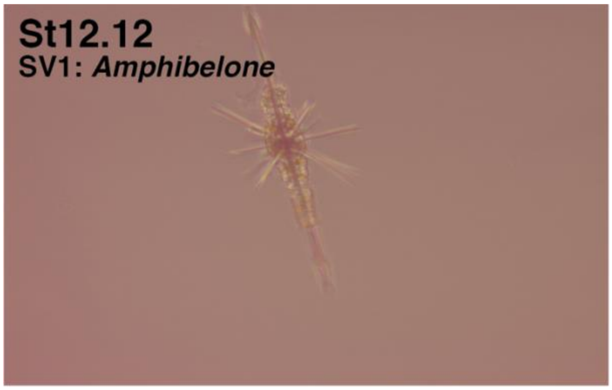
St12.12
SV1: *Amphibelone*

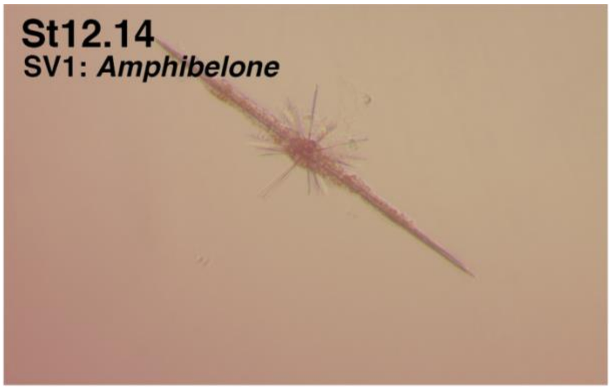
St12.14
SV1: *Amphibelone*

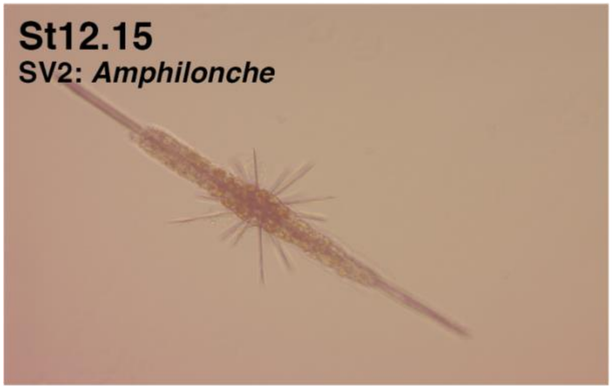
St12.15
SV2: *Amphilonche*

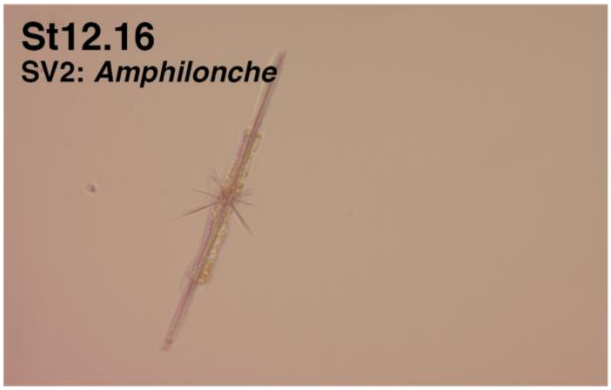
St12.16
SV2: *Amphilonche*

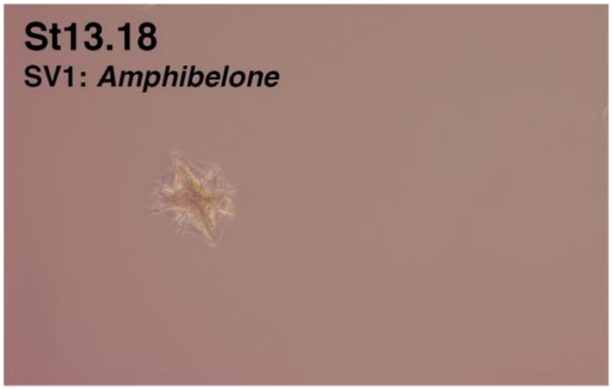
St13.18
SV1: *Amphibelone*

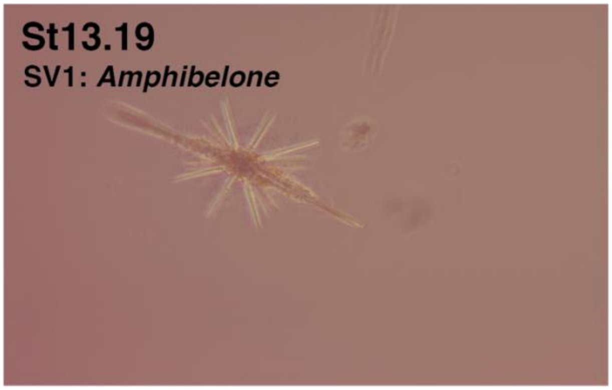
St13.19
SV1: *Amphibelone*

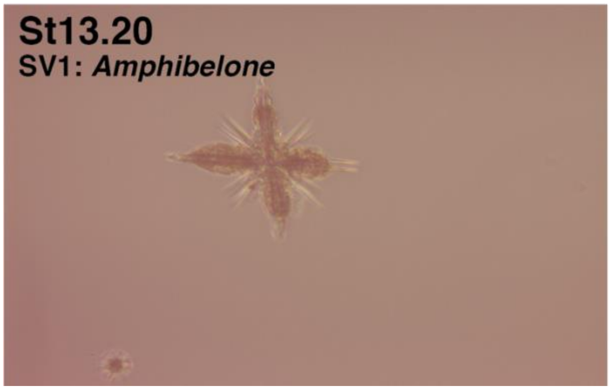
St13.20
SV1: *Amphibelone*

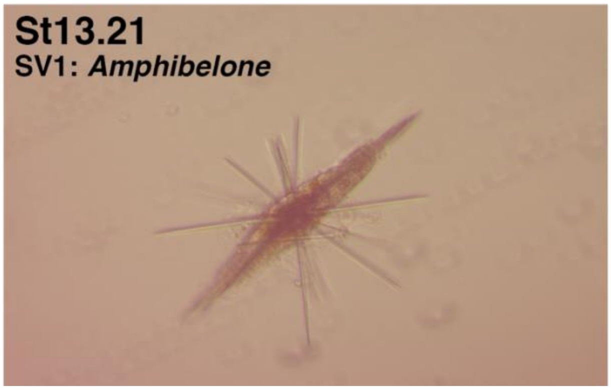
St13.21
SV1: *Amphibelone*

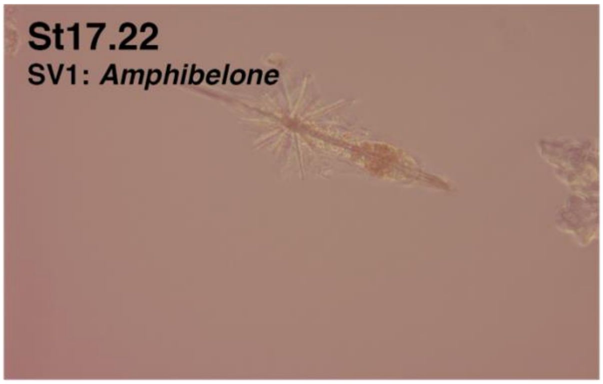
St17.22
SV1: *Amphibelone*

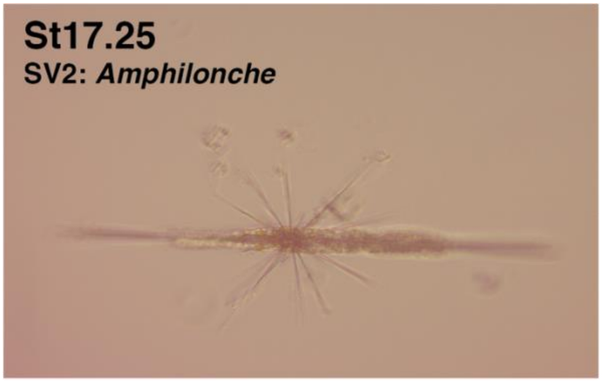
St17.25
SV2: *Amphilonche*

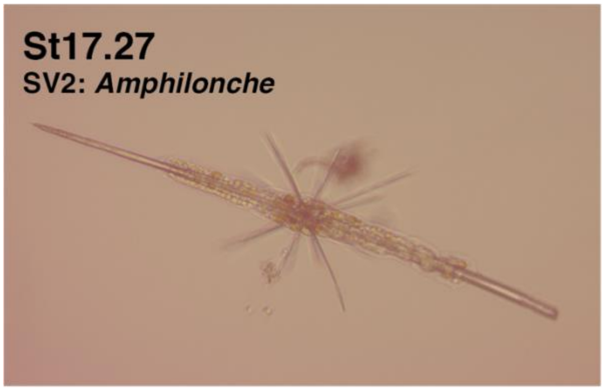
St17.27
SV2: *Amphilonche*

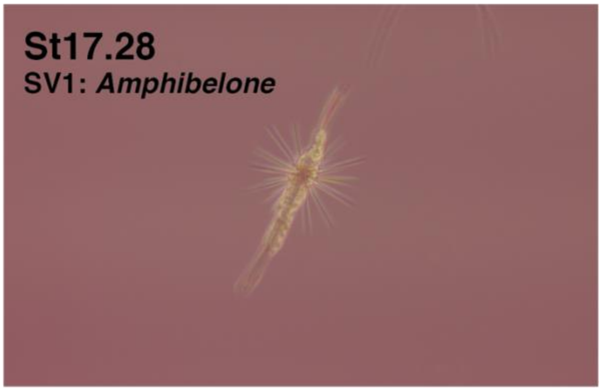
St17.28
SV1: *Amphibelone*

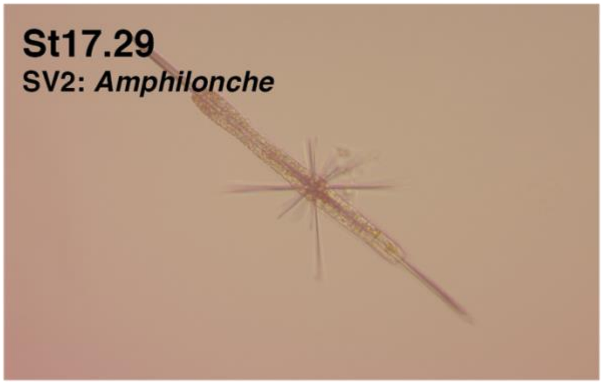
St17.29
SV2: *Amphilonche*

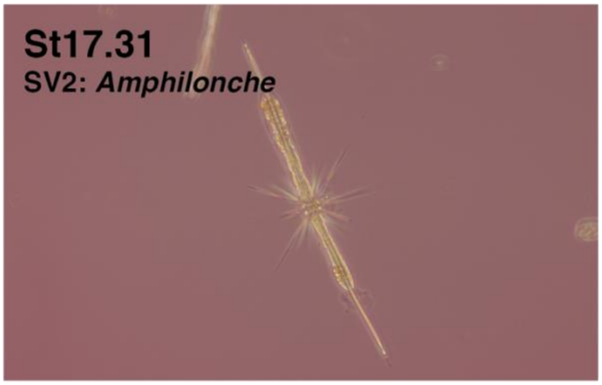
St17.31
SV2: *Amphilonche*

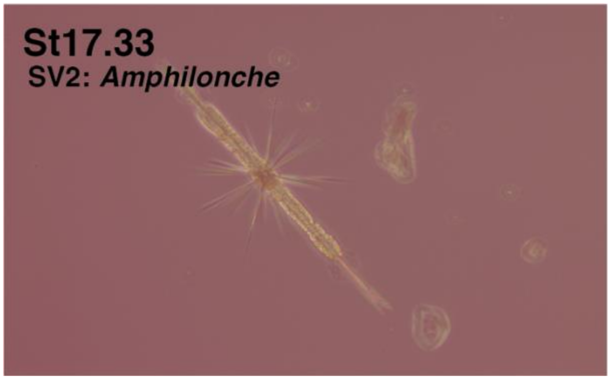
St17.33
SV2: *Amphilonche*

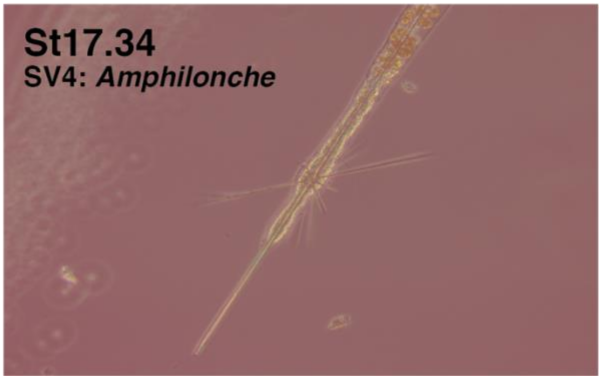
St17.34
SV4: *Amphilonche*

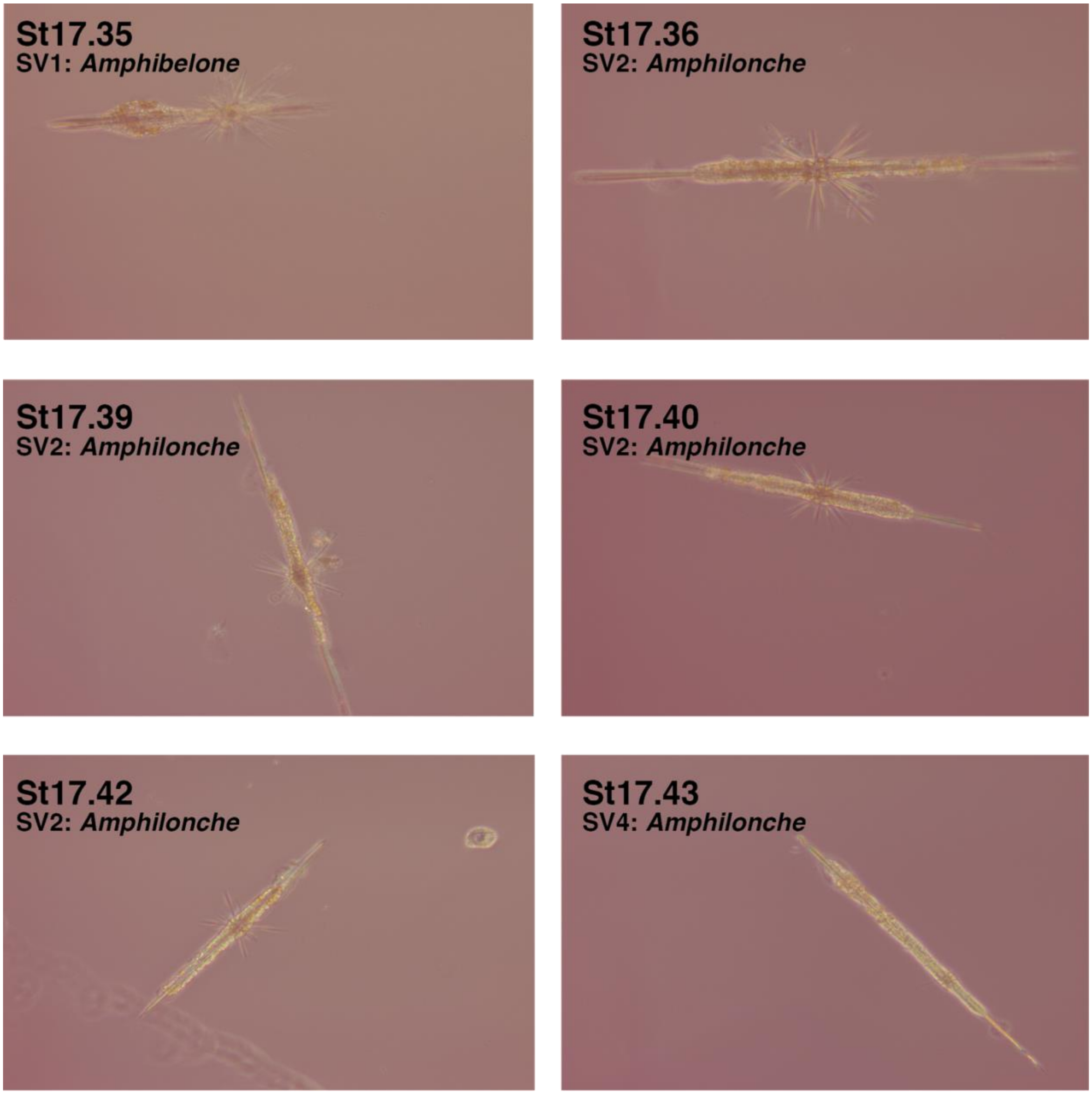

Supplemental Figure S2. Light microscopy images of acantharians collected from ECS cruise stations in May and June 2017. Samples are labeled by station number and sample number. The Host SV and the associated Acantharea genus is included under the sample name. All samples were imaged at 200x on a Zeiss PrimoVert inverted light microscope. Images are not cropped or otherwise distorted.

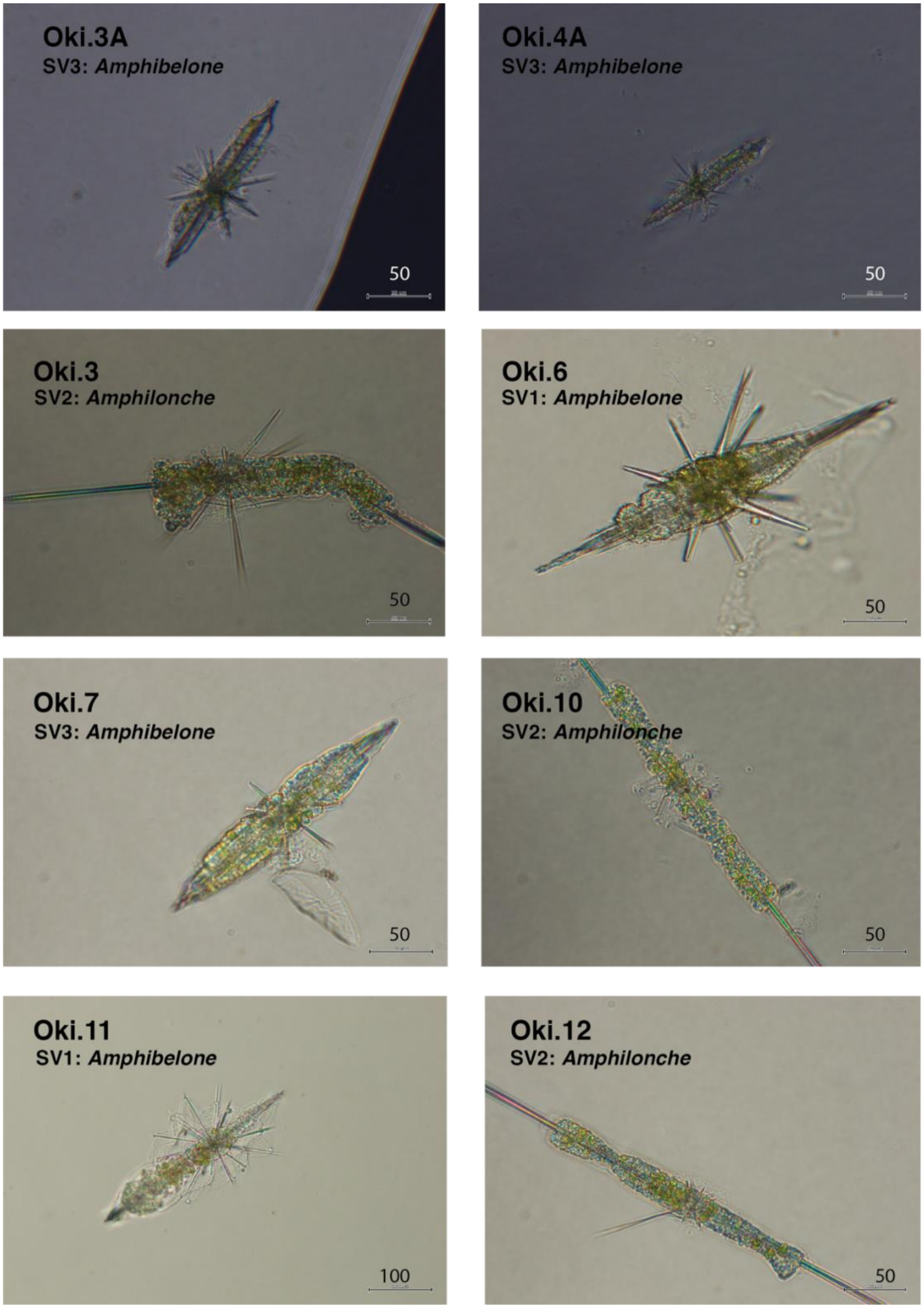

Supplemental Figure S3. Light microscopy images of acantharians collected near Okinawa in April (Oki.3A and Oki.4a) and May 2017 (Oki.3, 6, 7, 10, 11 & 12). The Host SV and associated Acantharea genus is indicated below the sample ID. Acantharians were imaged with an Olympus CKX53 inverted light microscope. Scale bars are in micrometers.

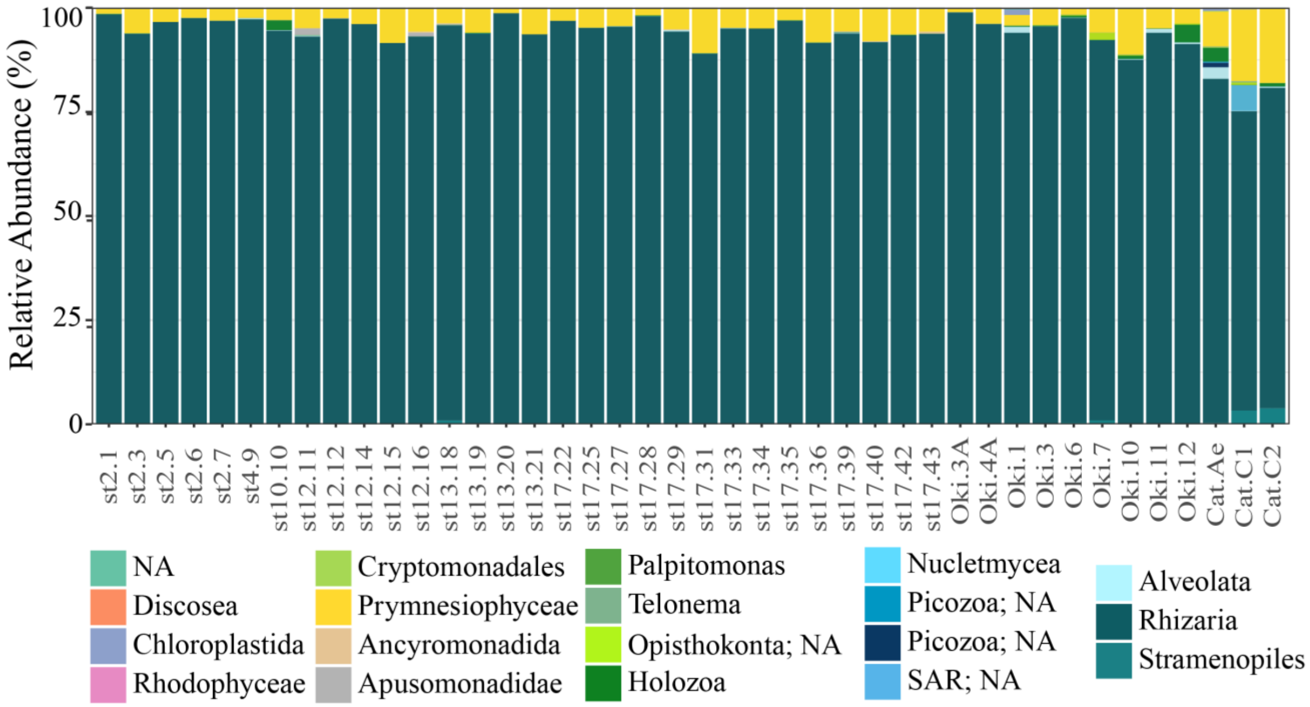

Supplemental Figure S4. Relative abundance of Sequence Variants (SVs) in single acantharian holobionts. Each bar represents a single acantharian host and is labeled by collection location (st#: ECS cruise station number, Oki: Okinawa Island, Cat: Catalina Island) and sample ID. Relative abundance includes all SVs remaining after initial prevalence filtering. Within each host, 72-99% of sequences were classified as Rhizaria and 1–17% of sequences were classified as Prymnesiophyceae.

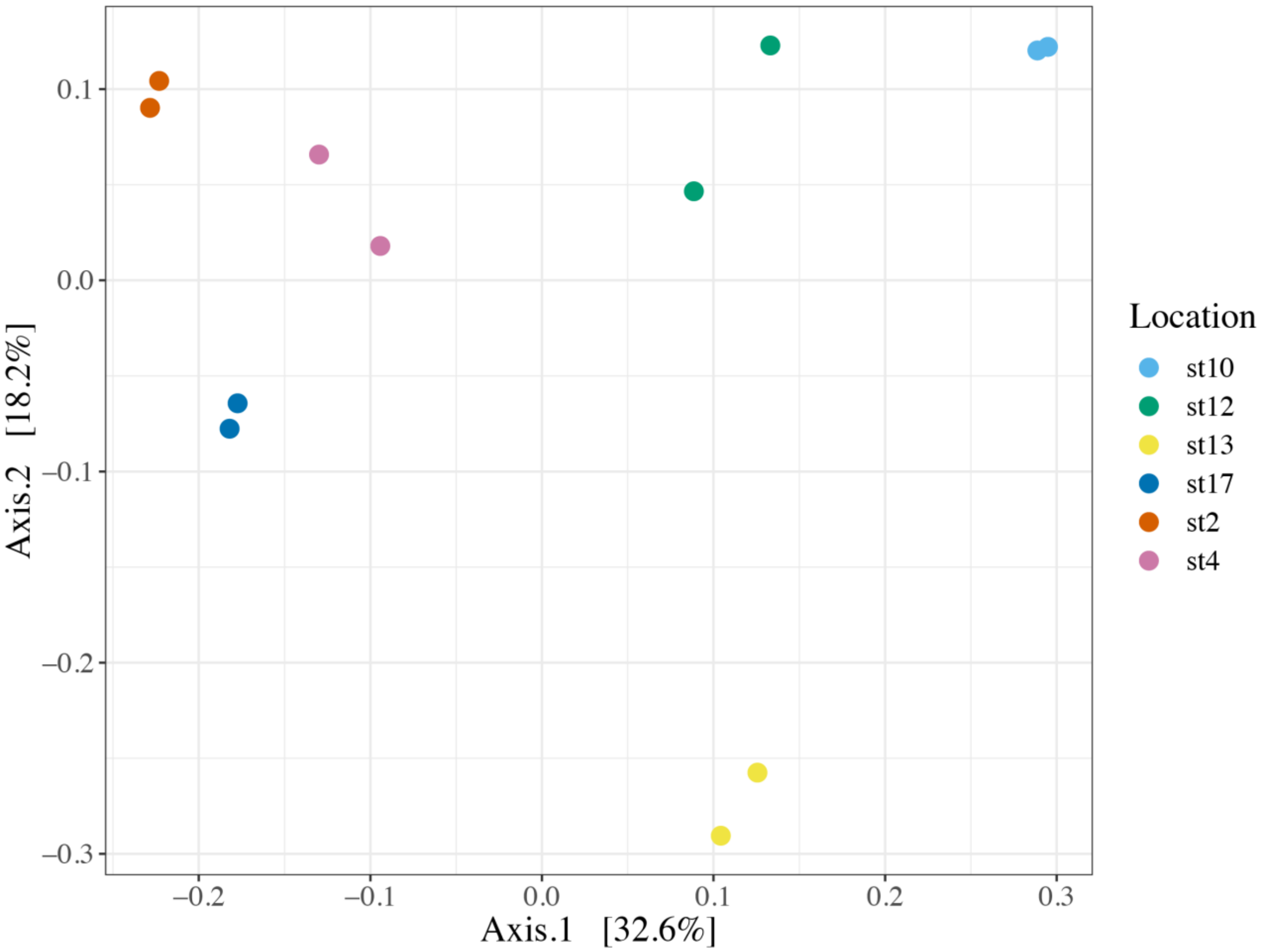

Supplemental Figure S5. Principal Coordinates Analysis of Bray-Curtis distances between microbial eukaryotic communities at East China Sea (ECS) cruise stations. Point color corresponds to the 6 ECS cruise stations where surface water samples were collected when collecting acantharians. Bray-Curtis distances between microbial eukaryote communities in ECS cruise station samples were calculated from entire microbial eukaryote communities in the greater than 0.2 μm and less than 10.0 μm size fraction**.** Biological replicates of environmental microbial eukaryote communities in each location are more similar to each other than to replicates from other locations. Bray-Curtis distance and PCoA ordination were performed with R package phyloseq and the plot was rendered with R package ggplot2

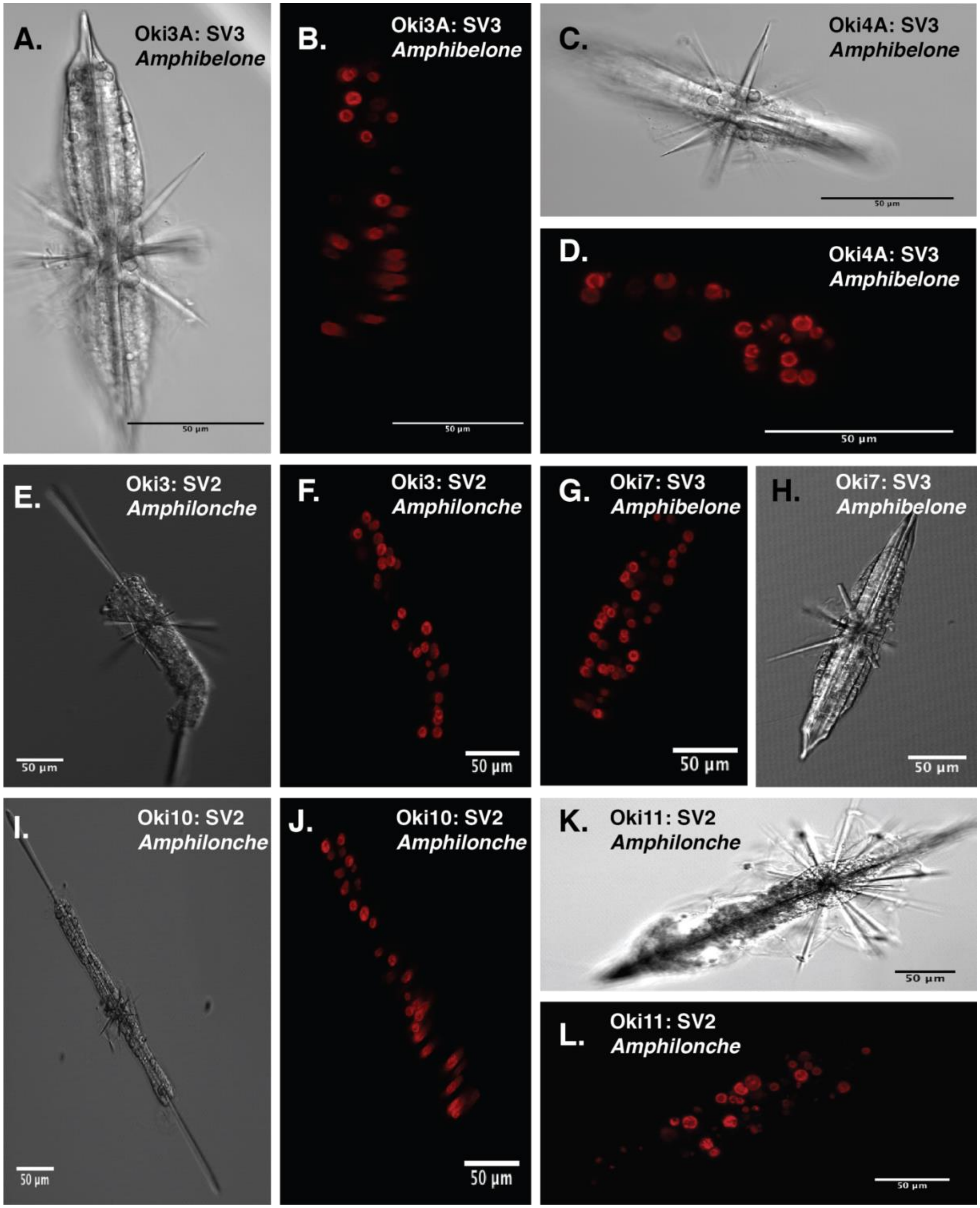

Supplemental Figure S6. Laser confocal microscopy of acantharians collected near Okinawa in April and May 2017. Acantharians were imaged with a Zeiss LSM780 inverted laser scanning confocal microscope. Halogen light images (A, C, E, H, I, K) are single optical slices and fluorescent images (B, D, F, G J, L) are maximum projections of z-stacks spanning the entire host, with symbiont chlorophyll autofluorescence colored red. Scale bars are 50 μm in all panels. Images are labeled by sample ID, host SV, and associated host genus.

2.2 Supplemental Tables

Location Name	Latitude	Longitude	Sampling Date	Time
Okinawa	26.446°	127.782°	4/14/17, 5/18/17, 12/16/17	9:00 AM
Catalina	33.449°	-118.488°	5/3/17	11:30 AM
St. 2	26.291°	126.4693°	5/30/17	9:30 AM
St. 4	25.933°	126.8994°	5/31/17	2:00 PM
St. 10	24.860°	123.8468°	6/3/17	10:15 AM
St. 12	27.788°	126.906°	6/4/17	1:30 PM
St. 13	29.006°	127.341°	6/5/17	9:00 AM
St. 17	28.959°	129.5685°	6/9/17	12:40 PM

Supplemental Table S1. GPS coordinates for sampling locations and date and time samples were collected.

Group 1 (Location)	Group 2 (Location)	Sample size	Permutation:	pseudo-F	p-value	q-value
Catalina	Okinawa	12	999	7.43164101	0.004	0.015
Catalina	st12	8	999	16.333752	0.015	0.03642857
Catalina	st13	7	999	10.5921875	0.034	0.04636364
Catalina	st17	17	999	11.616947	0.002	0.01
Catalina	st2	8	999	21.8721806	0.022	0.04125
Okinawa	st12	14	999	2.83989237	0.031	0.04636364
Okinawa	st13	13	999	0.48295534	0.78	0.78
Okinawa	st17	23	999	3.13559058	0.017	0.03642857
Okinawa	st2	14	999	8.86070735	0.001	0.0075
st12	st13	9	999	3.59513461	0.049	0.05653846
st12	st17	19	999	1.25981423	0.303	0.32464286
st12	st2	10	999	2.52812048	0.043	0.05375
st13	st17	18	999	3.1249681	0.033	0.04636364
st13	st2	9	999	9.43420133	0.006	0.018
st17	st2	19	999	5.2704721	0.001	0.0075

Supplemental Table S2. PERMANOVA results table for pair-wise comparisons by collection location. Numbered locations represent ECS cruise station numbers. Pairwise PERMANOVA (999 permutations) were performed with the beta-group-significance function in the Qiime2 diversity plugin.

Group 1 (Host SV)	Group 2 (Host SV)	Sample size	Permutations	pseudo-F	p-value	q-value
1	2	31	999	0.80869499	0.517	0.57444444
1	3	20	999	0.57101434	0.722	0.722
1	4	17	999	1.06666291	0.376	0.495
1	5	17	999	3.79294356	0.016	0.08
2	3	21	999	0.99948027	0.396	0.495
2	4	18	999	2.63794076	0.078	0.195
2	5	18	999	7.10086262	0.005	0.05
3	4	7	999	1.91998571	0.204	0.408
3	5	7	999	5.24113093	0.04	0.13333333
4	5	4	999	13.7019544	0.325	0.495

Supplemental Table S3. PERMANOVA results table for pair-wise comparisons by host SV. Pairwise PERMANOVA (999 permutations) were performed with the beta-group-significance function in the Qiime2 diversity plugin.
